# Community-Based Network Study of Protein-Carbohydrate Interactions in Plant Lectins Using Glycan Array Data

**DOI:** 10.1371/journal.pone.0095480

**Published:** 2014-04-22

**Authors:** Adeel Malik, Juyong Lee, Jooyoung Lee

**Affiliations:** Center for *In Silico* Protein Science, School of Computational Sciences, Korea Institute for Advanced Study, Seoul, Korea; University of South Florida College of Medicine, United States of America

## Abstract

Lectins play major roles in biological processes such as immune recognition and regulation, inflammatory responses, cytokine signaling, and cell adhesion. Recently, glycan microarrays have shown to play key roles in understanding glycobiology, allowing us to study the relationship between the specificities of glycan binding proteins and their natural ligands at the omics scale. However, one of the drawbacks in utilizing glycan microarray data is the lack of systematic analysis tools to extract information. In this work, we attempt to group various lectins and their interacting carbohydrates by using community-based analysis of a lectin-carbohydrate network. The network consists of 1119 nodes and 16769 edges and we have identified 3 lectins having large degrees of connectivity playing the roles of hubs. The community based network analysis provides an easy way to obtain a general picture of the lectin-glycan interaction and many statistically significant functional groups.

## Introduction

Glycans play important roles inside eukaryotic cells by binding to proteins and lipids, and they are also found in the extracellular space between cells [1]. Glycans can be grouped into two classes; linear sugars and polysaccharides. The polysaccharides consist of repeating pyranose monosaccharide rings and branched sugars, which are formed by linking various monosaccharide units [2]. Through non-covalent interactions with lectins, glycans control biochemical reactions by engaging in various biological processes such as development [3], [4], coagulation [5] and response to infection by bacterial and viral agents [6]. The size of the cellular glycome is believed to be in range of 100000–500000 glycans [7]. This large size of glycomic contents could be attributed to the combinatorial aspect that oligosaccharide chains come in either linear or branched form, monosaccharide building blocks are either in α or in β anomeric configurations and monosaccharides can be linked via various carbon atoms in their sugar rings [8]. Using the complexity of the glycome, cells adopt to encode a massive amount of biological information, and it is a great challenge to decode this hidden information to understand the biology of lectins and their interactions with carbohydrates.

Protein-carbohydrate interactions are involved in a variety of biological and biochemical processes, and, recently, attempts to understand the molecular basis of such interactions have appeared [9]. Traditional methods to probe glycan–protein recognition events include X-ray crystallography, NMR spectroscopy, the hemagglutination inhibition assay [10], enzyme-linked lectin assay [11], surface plasmon resonance [12] and isothermal titration calorimetry [13]. Although these methods have been successfully applied to elucidate the details of carbohydrate–protein interactions, they are rather labor intensive and require large amounts of carbohydrate samples. These shortcomings make the aforementioned traditional approaches unsuitable as high-throughput analytic methods [14]. On the other hand, recently, many computational methods have been suggested to study protein carbohydrate interactions [15]–[21].

Conventional methods for carbohydrate ligand detection are often cumbersome and we need sensitive and high-throughput technologies that can analyze carbohydrate-protein interactions in order to discover and differentiate oligosaccharide sequences interacting with carbohydrate binding proteins [8]. Carbohydrate micro-array based technology can serve as an appropriate method [22]–[25]. However, at present, one of the biggest limiting factors in utilizing the complete potential of the glycan microarray data is the lack of efficient analysis tools to extract relevant information.

For complete utilization of a glycan microarray data, we need a systematic computational method [26]. Large quantities of data are generated from the analysis of the Consortium for Functional Glycomics (CFG) glycan microarray [27]. Also, predicting the glycan-binding specificity or binding motif can be a time consuming step of scrutinizing and evaluating the linear sequences of monosaccharides in glycans [27]. The CFG offers glycan microarray data for various lectins (both plant and animal origin) and glycan binding antibodies. Recently computational methods have been developed for analyzing the glycan-binding specificity from glycan array data such as the motif-segregation method [26] and the outlier motif analysis (OMA) method [28].

In this work, we have developed a method to group various plant lectins and their interacting carbohydrates by the community detection analysis of a lectin-glycan network generated by the glycan microarray data from CFG. The lectin-glycan network consists of 1119 nodes (lectins and glycans) and 16769 edges (interactions). From this network, we have identified 3 lectins having large degrees of connectivity playing the roles of hubs. Additionally, we compared the results of our community detection method with other well known clustering algorithms. We show that our method outperforms existing clustering methods in terms of both modularity score as well as the number of statistically significant (p-value ≤0.05) glycan specific lectin groups. We propose that this study can reveal a global organization of lectin-glycan interactions, and help to identify strongly correlated lectin and glycan clusters.

## Methodology

### Data Generation

**Figure 1:**
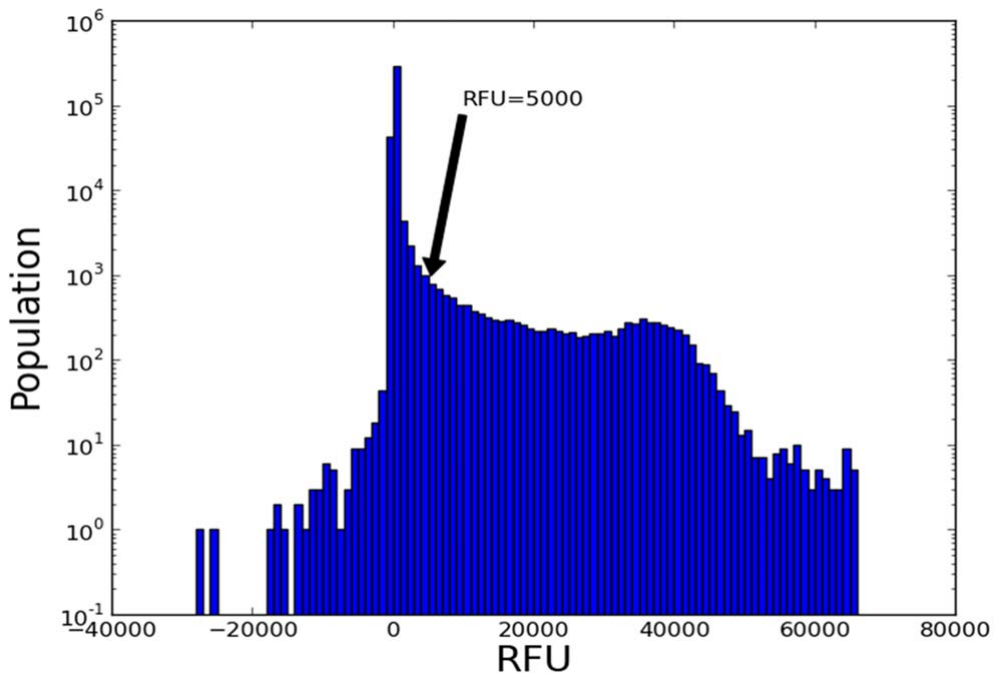
Histogram of the RFU values collected from 786 glycan array files is shown. It should be noted that the y-axis is shown in the log scale and the data corresponding to RFU larger than 5000 constitutes only about 3.5% of the whole data.

A total of 786 glycan array files for plant lectins were downloaded using a custom made script from Consortium for Functional Glycomics (CFG) as of Dec 2013. CFG provides extensive glycomics resources so that one can explore functions of glycans and glycan-binding proteins that play important roles in human health and disease [http://www.functionalglycomics.org/static/consortium/consortium.shtml]. All of these 786 files were further processed into a single input file, which consists of rows of protein-carbohydrate pairs. Three datasets were generated by filtering the protein-carbohydrate pairs using the cutoff values of relative fluorescence units (RFU) 5000, 10000 and 20000. These three datasets were used for network construction and their community detection. **Figure 1** shows the histogram of the RFU values collected from 786 glycan array files. The data corresponding to RFU larger than 5000 constitutes only about 3.5% of the whole data. All the data is available to researchers upon request.

### Network Construction

To perform a systematic analysis of protein-carbohydrate interaction, we have constructed a bipartite network, where unweighted edges are assigned between proteins and carbohydrates. Each node represents a lectin or a glycan and its identity is indicated by its array ID or glycan ID at a given condition. A glycan array ID represents a specific protein under a specific condition. Therefore, two different nodes in the network may represent two different concentrations of a protein in the glycan array experiment. The strength of a lectin-glycan interaction is represented by its RFU value and three networks are generated using three cutoff values of RFU of 5000, 10000 and 20000.

### Community Detection of a Network

We have identified the community structure of the lectin-glycan network by using the Mod-CSA method, which is a highly effective modularity optimization method [29], [30], [31]. The modularity is a widely used measure to determine the community structures of various networks. From a given community structure it measures the difference between the number of inter-community edges and its expected value from a randomly re-wired counterpart preserving the degrees of nodes. Modularity (Q) is defined as:
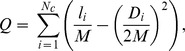
where *M* is the total number of edges in the network, 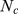 is the number of communities, 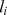 is the number of edges within community *i* and 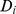 is the sum of degrees of nodes in community *i*. The value of *Q* ranges between −1 and 1 and it becomes close to 1 for a highly modular community structure and 0 for a random community structure [32].

### Network Visualization and Comparison with other Clustering Methods

Three lectin glycan array networks constructed in this study were exported to the Cytoscape 2.8.2, a bioinformatics package for biological network visualization and data integration [33]. To compare our clustering method with other widely used network clustering algorithms such as MCL [34], [35], MCODE [36] and greedy algorithm [32], we have used clusterMaker [37] and GLay plugins [38], a multi-algorithm clustering plugins for Cytoscape.

### Enrichment of Glycan-specific Proteins

Enriched glycan-specific lectins within each cluster were investigated by annotating each lectin with a predetermined glycan binding specificity. Reported specificities of various lectins were extracted from literature [39], [40] and Uniprot database [41] as summarized in **Table 1** **.** The full list of all 513 protein nodes used in this study with annotations (wherever possible) are listed in **Table S1**.

**Table 1:** List of glycan binding specificities of lectins investigated in this study is shown. Specificities are collected from literature and uniprot database.

S. No.	Protein Name	Reported Specificity
1.	Pokeweed Agglutinin	(GlcNAcb1-4)n
2.	Datura Stramonium Lectin	(GlcNAcb1-4)n, Galb1-4GlcNAc
3.	Soybean Agglutinin	a- or b-linked terminal GalNAc, GalNAca1-3Gal
4.	LBA Lima Bean Agglutinin/LBL	a-D-GalN.Ac
5.	Griffonia Simplicifolia Lectin I, Isolectin B4/GSI-B4 isolectin	a-Linked Gal
6.	Agglutinin	a-Linked terminal GalNAc
7.	Psophocarpus tetragonolobus Agglutinin/Basic agglutinin	a-Linked terminal GalNAc
8.	Psophocarpus Tetragonolobus Lectin I	a-Linked terminal GalNAc
9.	Vicia Villosa Lectin (VVL)	a-Linked terminal GalNAc, GalNAca1-3Gal
10.	Griffonia simplicifolia II/InsecticidalN-acetylglucosamine-specific lectin	Agalactosylated tri/tetra antennary glycans, GlcNAc
11.	Phaseolus vulgaris Erythroagglutinin/Erythroagglutinating phytohemagglutinin	Bi-antennary complex-type N-glycan with outer Galand bisecting GlcNAc
12.	Wheat Germ Agglutinin (WGA)	Chitin oligomers, Sia
13.	Laburnum alpinum Agglutinin/Lectin 1/Seed lectin anti-H(O)	Di-N-acetylchitobiose specific lectin.
14.	Ulex europaeus AgglutininII/UEA-II OR Anti-H(O) lectin 2	Di-N-acetylchitobiose specific lectin.
15.	Trichosanthes japonica Agglutinin II	Fuca1-2Galb1 -> or GalNAcb1 -> groups attheir nonreducing terminals
16.	Cholera Toxin B	Fuca1-2Galb1-3GalNAcb1-4(Neu5Aca2-3)Galb1-4Glcb ORGalb1-3GalNAcb1-4(Neu5Aca2-3)Galb1-4Glcb
17.	Ulex Europaeus Agglutinin ORAnti-H(O) lectin 1	Fuca1-2Galb1-4GlcNAc
18.	Lotus Tetragonolobus Lectin/Anti-H(O) lectin	Fuca1-3(Galb1-4)GlcNAc, Fuca1-2Galb1-4GlcNAc
19.	Aspergillus oryzae Lectin	Fuca1-6GlcNAc (core fucose)
20.	Lens Culinaris Agglutinin	Fuca1-6GlcNAc, a-D-Glc, a-D-Man
21.	Pisum Sativum Agglutinin	Fuca1-6GlcNAc, a-D-Glc, a-D-Man
22.	Aleuria Aurantia Lectin AAL	Fuca1-6GlcNAc, Fuca1-3(Galb1-4)GlcNAc
23.	Pseudomonas aeruginosa lectin/PA-I galactophilic lectin	Fucose Anywhere
24.	Psophocarpus Tetragonolobus Lectin II	Fucose binding lectin
25.	Fucose-binding lectin protein	Fucose binding lectin
26.	Euonymus europaeus Agglutinin	Gala1-3Gal, blood group B antigen
27.	Cytisus sscoparius Agglutinin	Galactose binding lectin
28.	Discoidin-2	Galactose binding lectin
29.	Polyporus Squamosus Lectin	Galactose binding lectin
30.	Discoidin-1 subunit B/C	Galactose- and N-acetylgalactosamine-binding
31.	SRL- strong binding to di-saccharideGalb1!3GalNAc-a- similar to Agaricus bisporuslectin	Galb1->3GalNAc-a-
32.	Agaricus bisporus Agglutinin	Galb1-3GalNAc
33.	Amaranthus Caudatus Lectin	Galb1-3GalNAc
34.	Galactose-binding lectin (Agglutinin PNA)	Galb1-3GalNAc
35.	Jacalin/Agglutinin alpha chain	Galb1-3GalNAc, GalNAc
36.	Bauhinia Purpurea Lectin	Galb1-3GalNAc, GalNAc
37.	Maclura Pomifera Lectin/Agglutinin alpha chain/MPA	Galb1-3GalNAc, GalNAc
38.	Erythrina crista-galli Lectin	Galb1-4GlcNAc
39.	Ricinus Communis Agglutinin I	Galb1-4GlcNAc
40.	Dolichos biflorus Agglutinin/Seed lectin subunit I	GalNAca1-3GalNAc, blood group A antigen
41.	Wisteria floribunda Agglutinin	GalNAcb1-4GlcNAc, Galb1-3(-6)GalNAc
42.	Marasmium oreades agglutinin	Galα(1,3)Gal
43.	Solanum Tuberosum (Potato) Lectin (STL)	GlcNAc oligomers, oligosaccharide containingGlcNAc and LacNAc
44.	Lycopersicon Esculentum Lectin	GlcNAc trimers/tetramers
45.	Urtica dioica Agglutinin/Lectin/endochitinase 1	GlcNAcb1-4GlcNAc, Mixture of Man5–Man9
46.	Coprinopsis cinerea lectin 2	GlcNAcβ1,4[Fucα1,3]GlcNAc
47.	Vicia faba Agglutinin	Glucose binding lectin
48.	Galanthus nivalis agglutinin orMannose-specific lectin	High-mannose, Mana1-3Man
49.	Hippeastrum hybrid Agglutinin	High-mannose, Mana1-3Man, Mana1-6Man
50.	Canavalia A (Con A)	High-mannose, Mana1-6(Mana1-3)Man
51.	Canavalia ensiformis (Con A)	High-mannose, Mana1-6(Mana1-3)Man
52.	Narcissus pseudonarcissusAgglutinin	High-mannose, Mana1-6Man
53.	Tulip Lectin	Mana1-3(Mana1-6)Man, bi- and tri-antennarycomplex-type N-glycan, GalNAc
54.	Sauromatum gutattum	Manb Anywhere
55.	Mannose specific lectin	Mannose binding lectin
56.	ASA, Allium sativum agglutinin(ASAI and ASAII)	Mannose binding lectin
57.	Lectin	Mannose binding lectin
58.	Concanavalin-A	Mannose binding lectin
59.	Colocasia esculenta Lectin	Mannose binding lectin
60.	Lectin alpha chain	Mannose binding lectin
61.	Mannose-binding lectin	Mannose binding lectin
62.	Banana lectin	Mannose binding lectin
63.	Cyanovirin-N	Mannose binding lectin
64.	Salt stress-induced protein	Mannose binding lectin
65.	Lectin-like protein	Mannose binding lectin
66.	Hessian fly response gene 1 protein	Mannose binding lectin
67.	Nessun dorma, isoform A;Nessun dorma, isoform B	N-acetylglucosamine
68.	Nicotiana tabacum agglutinin	N-acetylglucosamine
69.	Psathyrella velutina lectin	N-acetylglucosamine and N-acetylneuraminic acid
70.	Ricin B-like lectin	N,N'-diacetyllactosediamine(GalNAcβ1-4GlcNAc, LacdiNAc)
71.	Maackia Amurensis Lectin II	Siaa2-3Galb1-
72.	Maackia Amurensis Lectin I	Siaa2-3Galb1-
73.	Maackia amurensis Agglutinin	Siaa2-3Galb1-3(Siaa2-6)GalNAc
74.	Sambucus nigra Agglutinin	Siaa2-6Gal/GalNAc
75.	Trichosanthes japonica Agglutinin I	Siaa2-6Gal/GalNAc
76.	Limax flavus Agglutinin/Sialic acid-binding lectin 1	Sialic acid-binding lectin
77.	Platypodium elegans legume lectin	Subterminal Mannose
78.	Sclerotinia sclerotiorumagglutinin	terminal N-acetylgalactosamine (GalNAc)
79.	Phaseolus vulgaris Leucoagglutinin/Leucoagglutinating phytohemagglutinin	Tri/tetra-antennary complex-type N-glycan

The enrichment of glycan-specificities of lectins in each cluster was assessed by calculating the hypergeometric p-value. The p-value corresponds to the probability that a given lectin cluster sharing the same glycan-specificity can be obtained by chances. The p-value was calculated as follows:
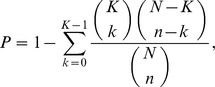
where *N* is the total number of lectins in the network, *K* is the number of all lectins having a particular glycan-specificity, and *k* is the number of lectins having the particular glycan-specificity in a cluster with the size of *n*.

Enrichment analysis was also attempted by using DAVID functional annotation cluster tool [http://david.abcc.ncifcrf.gov/home.jsp], which did not yield any statistical significant clusters. We then manually searched each lectin in InterPro database [42] but only 8 unique GO terms such as chitin-binding, carbohydrate-binding, protein binding, endopeptidase inhibitor activity, etc, were retrieved. However, these GO terms are too general to signify any detailed glycan binding specificities of corresponding lectins. Therefore, in this study, the enrichment analysis for each cluster was performed based on the annotations listed in **Table 1**. Only those clusters with at least 10 protein nodes were analyzed for statistical significance.

### Identification of Hub Proteins

**Figure 2:**
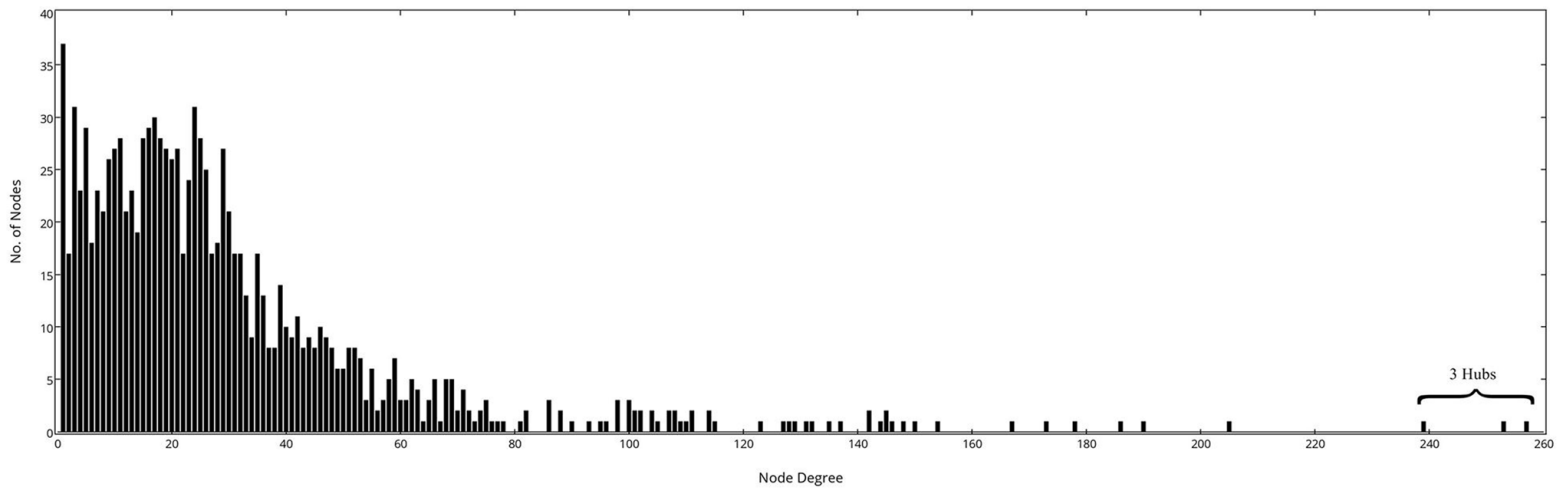
The node degree distribution of the lectin-glycan network is shown. We observe a large gap between 3 hub nodes and the other nodes. The degree distribution was plotted using plotly [https://plot.ly/plot].

In general, biological networks possess the scale-free property [43] in which only a few nodes in the network have many connections serving as hubs in the network. Hub proteins were identified by calculating the node degree distribution [44] by using the NetworkAnalyzer plugin of Cytoscape. Top three highest degree protein nodes were assigned as hubs (see **Figure 2**).

## Results and Discussion

**Figure 3:**
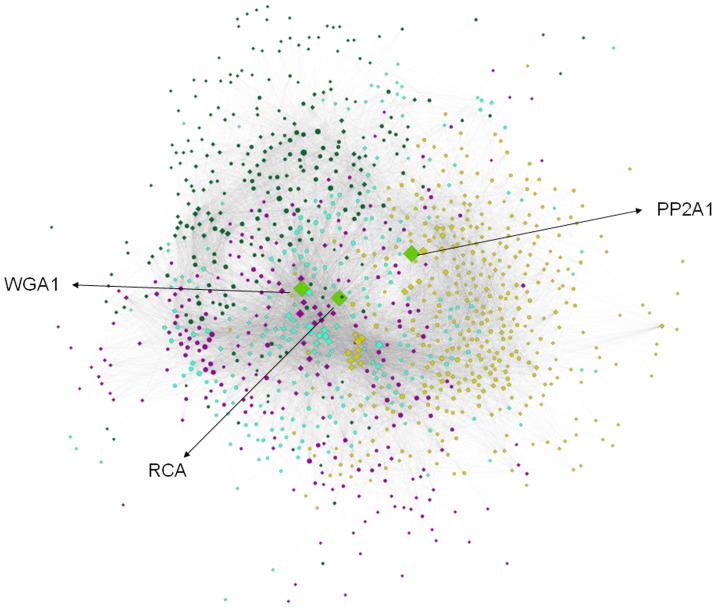
The lectin-glycan network generated using the RFU cut-off of 5000 is shown. Circles represent glycan nodes and diamonds represent lectin nodes. The nodes are color coded according to their communities. Three hub nodes (shown in green diamonds) are PP2A1, WGA1 and RCA.

We constructed three lectin-glycan interaction networks by using the plant lectin-glycan micro array data filtered by three RFU cut-offs. The network where the interactions were filtered by RFU <5000 consists of 1119 nodes (513 proteins and 606 carbohydrates) and 16769 edges. Similarly, the second network filtered by RFU <10000 has 1035 nodes and 12169 edges, and the third one (filtered by RFU <20000) consists of 901 nodes and 8042 edges. Since the first network has the maximum number of nodes and edges, and shows more statistically significant glycan specific groups (discussed later) than the other two networks, the results specified henceforth represent the first network if not specifically indicated. The first network is shown in **Figure 3**, where proteins are represented as diamonds and glycans as circles and the interactions between them are represented as edges.

The network representation enables a quick visual inspection of the glycans bound to a lectin of interest. Additionally, in order to identify hub lectins from the lectin-glycan array, the node degree distribution of the network was calculated and is shown in **Figure 2**. In an interaction network, proteins that interact with a large number of partners are considered as hubs [45], and are essential components of biological networks [46]. The definition of the hub node is rather subjective, but based on the observation of the biggest gap between the 3^rd^ and 4^th^ largest degree nodes in **Figure 2**, we assigned hub proteins as those three with degree larger than 220. The 3 hubs are Phloem Protein2 (PP2A1) from Arabidopsis thaliana, wheat germ agglutinin (WGA) from Triticum vulgaris (wheat), and Ricinus communis agglutinin (RCA) from Ricinus communis (castor bean).

By using the Mod-CSA method, the lectin-glycan network is clustered into 4 modules (communities), which are represented by separate colors in **Figure 3**. The largest module consists of 168 protein nodes and 215 glycan nodes, and the smallest community contains 98 protein nodes and 133 glycan nodes.

To validate the lectin-glycan interaction network and its detected community-structure, we investigated the binding specificities of the first neighbors of two plant lectins, Sambucus nigra agglutinin (SNA) and concanavalin A (ConA) whose glycan binding specificities are well known. The first lectin is a well-characterized plant lectin, elderberry bark agglutinin from Sambucus nigra, which is known to recognize the Neu5Aca2-6Gal linkage [47]. The second one is concanavalin A (ConA), which is known to have specificity for mannose sugars [48], [49], [50]. Proper categorization of the specificities of glycan-binding proteins plays a significant role in understanding protein-glycan interactions and utilizing glycan-binding proteins as analytical reagents.

### Binding Specificities of SNA

It is well known that some plants contain more than one lectin with different sugar binding specificities [51]. The bark of the elderberry (*Sambucus nigra)* has two lectins SNA-I and SNA-II with different glycan binding specificities. *Sambucus nigra agglutinin* I (SNA-I), is the first lectin identified from the elderberry bark which has been conventionally employed to recognize Neu5Acα2-6Gal [47] or Neu5Acα2-6Galβ1-4GlcNAc sequence [27]. SNA-I is composed of two polypeptides, namely chain A of 33 kDa with enzymatic activity, and chain B of 35 kDa with carbohydrate-binding activity [52]. Molecular modeling studies have indicated that the overall structure of SNA-I is quite similar to that of Ricin [53] and SNA-I belongs to the group of type 2 ribosome-inactivating proteins [52]. SNA-II is the second lectin isolated from the elderberry bark tissue, and it exhibits high affinity for glycoconjugates and Type 14 pneumococcal polysaccharides having multiple terminal D-Gal groups [51]. SNA-II consists of two identical carbohydrate-binding B-chains [51], [52].

In the current lectin glycan array network, nineteen nodes represent both SNA-I and SNA-II lectins. Out of these nineteen SNA nodes, fifteen SNA-I nodes are from community 1 (1000180, 1000181, 1000183, 1000184 and 1000725), and community 3 (1002793, 1004421, 1004422, 1004701, 1004702, 1004703, 1004704, 1004705, 1004706 and 1004780). Similarly, SNA-II is represented by four nodes (1004707, 1004708, 1004709 and 1004710) enriched in community 3.

The 10 SNA-I nodes in community 3 show specificity for complex-type biantennary N-glycans (**Table 2A**). From this table we observe that almost all of the interacting glycans possess the determinant **Neu5Acα2-6Gal** or **Neu5Acα2-6Galβ1-4GlcNAc** (shown by bold text in the table). Another interesting point to notice is that the glycans 527 and 479 exhibit low RFU values in **Table 2**. This could be due to the fact that these glycans contain **Neu5Acα2-3** sequence, which is known to decrease the binding of SNA [27]. On the other hand, 316 (Neu5Acα2-3Galβ1-4GlcNAcβ1-2Manα1-3(Neu5Acα2-6Galβ1-4GlcNAcβ1-2Manα1-6)Manβ1-4GlcNAcβ1-4GlcNAcβ-Sp12) contains two sequences, one (**Neu5Acα2-6Galβ1-4GlcNAc**) increasing the binding and the other (**Neu5Acα2-3**) decreasing the binding.

**Table 2:** Three types of complex glycans for SNA proteins are listed.

A)		
Glycan No.	Glycan Name	Avg. RFU
268	Neu5Acα2-6Galβ1-4(6S)GlcNAcβ-Sp8	51134
467	**Neu5Acα2-6Galβ1-4GlcNAc**β1-6(**Neu5Acα2-6Galβ1-4GlcNAc**β1-2)Manα1-6(GlcNAcβ1-4)(**Neu5Acα2-6Galβ1-4GlcNAc**β1-4(**Neu5Acα2-6Galβ1-4GlcNAc**β1-2)Manα1-3)Manβ1-4GlcNAcβ1-4GlcNAcβ-Sp21	48246
465	**Neu5Acα2-6Galβ1-4GlcNAc**β1-4Manα1-6(GlcNAcβ1-4)(**Neu5Acα2-6Galβ1-4GlcNAc**β1-4(**Neu5Acα2-6Galβ1-4GlcNAc**β1-2)Manα1-3)Manβ1-4GlcNAcβ1-4GlcNAcβ-Sp21	43978
346	**Neu5Acα2-6Galβ1-4GlcNAc**β1-2Manα1-6(Manα1-3)Manβ1-4GlcNAcβ1-4GlcNAc-Sp12	43812
327	**Neu5Acα2-6Galβ1-4GlcNAc**β1-3Galβ1-4GlcNAcβ1-3Galβ1-4GlcNAcβ-Sp0	41668
320	Galβ1-4GlcNAcβ1-2Manα1-6(**Neu5Acα2-6Galβ1-4GlcNAc**β1-2Manα1-3)Manβ1-4GlcNAcβ1-4GlcNAcβ-Sp12	41588
302	**Neu5Acα2-6Galβ1-4GlcNAc**β1-2Manα1-6(Galβ1-4GlcNAcβ1-2Manα1-3)Manβ1-4GlcNAcβ1-4GlcNAcβ-Sp12	41500
483	**Neu5Acα2-6Galβ1-4GlcNAc**β1-2Manα1-6(**Neu5Acα2-6Galβ1-4GlcNAc**β1-2Manα1-3)Manβ1-4GlcNAcβ1-4(Fucα1-6)GlcNAcβ-Sp24	41106
55	**Neu5Acα2-6Galβ1-4GlcNAc**β1-2Manα1-6(**Neu5Acα2-6Galβ1-4GlcNAc**β1-2Manα1-3)Manβ1-4GlcNAcβ1-4GlcNAcβ-Sp12	40488
348	**Neu5Acα2-6Galβ1-4GlcNAc**β1-2Manα1-6Manβ1-4GlcNAcβ1-4GlcNAc-Sp12	39574
606	**Neu5Acα2-6Galβ1-4GlcNAc**β1-3Galβ1-4GlcNAcβ1-6(**Neu5Acα2-6Galβ1-4GlcNAc**β1-3Galβ1-4GlcNAcβ1-3)GalNAca-Sp14	39290
482	Neu5Acα2-6Galβ1-4 GlcNAcβ1-6(**Neu5Acα2-6Galβ1-4GlcNAc**β1-3)GalNAca-Sp14	39202
57	**Neu5Acα2-6Galβ1-4GlcNAc**β1-2Manα1-6(**Neu5Acα2-6Galβ1-4GlcNAc**β1-2Man-a1-3)Manβ1-4GlcNAcβ1-4GlcNAcβ-Sp21	38592
56	**Neu5Acα2-6Galβ1-4GlcNAc**β1-2Manα1-6(**Neu5Acα2-6Galβ1-4GlcNAc**β1-2Manα1-3)Manβ1-4GlcNAcβ1-4GlcNAcβ-Sp13	37417
609	**Neu5Acα2-6Galβ1-4GlcNAc**β1-3Galβ1-4GlcNAcβ1-2Manα1-6(**Neu5Acα2-6Galβ1-** **4GlcNAc**β1-3Galβ1-4GlcNAcβ1-2Manα1-3)Manβ1-4GlcNAcβ1-4GlcNAcβ-Sp12	36652
457	**Neu5Acα2-6Galβ1-4GlcNAc**β1-6(Fucα1-2Galβ1-3GlcNAcβ1-3)Galβ1-4Glc-Sp21	36616
325	**Neu5Acα2-6Galβ1-4GlcNAc**β1-3Galβ1-3GlcNAcβ-Sp0	36221
314	**Neu5Acα2-6Galβ1-4GlcNAc**β1-2Manα1-3(Galβ1-4GlcNAcβ1-2Manα1-6)Manβ1-4GlcNAcβ1-4GlcNAcβ-Sp12	35848
503	Neu5Acα2-6GalNAcβ1-4(6S)GlcNAcβ-Sp8	33405
298	(6S)Galβ1-4(6S)GlcNAcβ-Sp0	32632
287	Neu5Gcα2-6Galβ1-4GlcNAcβ-Sp0	31718
354	Galβ1-4GlcNAcβ1-2Manα1-6(Galβ1-4GlcNAcβ1-2Manα1-3)Manβ1-4GlcNAcβ1-4(Fucα1-6)GlcNAcβ-Sp22	30544
557	Neu5Gcα2-8Neu5Gcα2-6Galβ1-4GlcNAc-Sp0	28273
366	Fucα1-4(Galβ1-3)GlcNAcβ1-2Manα1-6(Fucα1-4(Galβ1-3)GlcNAcβ1-2Manα1-3)Manβ1-4GlcNAcβ1-4(Fucα1-6)GlcNAcβ-Sp22	27993
319	**Neu5Acα2-6Galβ1-4GlcNAc**β1-2Manα1-6(Neu5Aca2-3Galβ1-4GlcNAcβ1-2Manα1-3)Manβ1-4GlcNAcβ1-4GlcNAcβ-Sp12	27611
54	**Neu5Acα2-6Galβ1-4GlcNAc**β1-2Manα1-3(**Neu5Acα2-6Galβ1-4GlcNAc**β1-2Manα1-6)Manβ1-4GlcNAcβ1-4GlcNAcβ-N(LT)AVL	27447
321	**Neu5Acα2-6Galβ1-4GlcNAc**β1-2Manα1-3(Neu5Aca2-3Galβ1-4GlcNAcβ1-2Manα1-6)Manβ1-4GlcNAcβ1-4GlcNAcβ-Sp12	26481
274	Neu5Acα2-6Galβ1-4Glcb-Sp8	25380
53	**Neu5Acα2-6Galβ1-4GlcNAc**β1-2Manα1-3(**Neu5Acα2-6Galβ1-4GlcNAc**β1-2Manα1-6)Manβ1-4GlcNAcβ1-4GlcNAcβ-Sp12	25345
48	[9NAc]**Neu5Acα2-6Galβ1-4GlcNAc**β-Sp8	21953
488	**Neu5Acα2-6Galβ1-4GlcNAc**β1-6(Fucα1-2Galβ1-4(Fucα1-3)GlcNAcβ1-3)Galβ1-4Glc-Sp21	21783
328	**Neu5Acα2-6Galβ1-4GlcNAc**β1-3Galβ1-3GlcNAcβ-Sp0	21014
324	**Neu5Acα2-6Galβ1-4GlcNAc**β1-2Manα1-3(Neu5Acα2-3Galβ1-4GlcNAcβ1-2Manα1-6)Manβ1-4GlcNAcβ1-4GlcNAcβ-Sp12	19830
58	**Neu5Acα2-6Galβ1-4GlcNAc**β1-2Manα1-6(**Neu5Acα2-6Galβ1-4GlcNAc**β1-2Manα1-3)Manβ1-4GlcNAcβ1-4GlcNAcβ-Sp24	18639
347	Manα1-6(**Neu5Acα2-6Galβ1-4GlcNAc**β1-2Manα1-3)Manβ1-4GlcNAcβ1-4GlcNAc-Sp12	16329
464	**Neu5Acα2-6Galβ1-4GlcNAc**β1-2Manα1-6(GlcNAcβ1-4)(**Neu5Acα2-6Galβ1-** **4GlcNAc**β1-2Manα1-3)Manβ1-4GlcNAcβ1-4GlcNAcβ-Sp21	16237
466	**Neu5Acα2-6Galβ1-4GlcNAc**β1-6(**Neu5Acα2-6Galβ1-4GlcNAc**β1-2)Manα1-6(GlcNAcβ1-4)(**Neu5Acα2-6Galβ1-** **4GlcNAc**β1-2Manα1-3)Manβ1-4GlcNAcβ1-4GlcNAcβ-Sp21	13858
409	Neu5Acα2-6Galβ1-3GlcNAcβ1-3(Galβ1-4GlcNAcβ1-6)Galβ1-4Glc-Sp21	12386
270	**Neu5Acα2-6Galβ1-4GlcNAc**β-Sp8	11415
317	**Neu5Acα2-6Galβ1-4GlcNAc**β1-2Manα1-3(Galβ1-4GlcNAcβ1-2Manα1-6)Manβ1-4GlcNAcβ1-4GlcNAcβ-Sp12	11124
360	KDNa2-3Galβ1-3GalNAca-Sp14	11019
485	Manα1-6(Manα1-3)Manβ1-4GlcNAcβ1-4(Fucα1-6)GlcNAcβ-Sp19	10968
427	Fucα1-2Galβ1-3GlcNAcβ1-2Manα1-6(Fucα1-2Galβ1-3GlcNAcβ1-2Manα1-3)Manβ1-4GlcNAcβ1-4(Fucα1-6)GlcNAcβ-Sp22	10833
458	**Neu5Acα2-6Galβ1-4GlcNAc**β1-6(Fucα1-2Galβ1-3GlcNAcβ1-3)Galβ-4Glc-Sp21	10202
52	**Neu5Acα2-6Galβ1-4GlcNAc**β1-2Manα1-3(**Neu5Acα2-6Galβ1-4GlcNAc**β1-2Manα1-6)Manβ1-4GlcNAcβ1-4GlcNAcβ-Sp8	9467
309	**Neu5Acα2-6Galβ1-4GlcNAc**β1-2Manα1-6(GlcNAcβ1-2Manα1-3)Manβ1-4GlcNAcβ1-4GlcNAcβ-Sp12	9381
376	**Neu5Acα2-6Galβ1-4GlcNAc**β1-3GalNAc-Sp14	8974
521	**Neu5Acα2-6Galβ1-4GlcNAc**β1-2Man-Sp0	8470
313	Neu5Acα2-3Galβ1-4GlcNAcβ1-2Manα1-3(**Neu5Acα2-6Galβ1-4GlcNAc**β1-2Manα1-6)Manβ1-4GlcNAcβ1-4GlcNAcβ-Sp12	8322
316	***Neu5Acα2-3Gal***β1-4GlcNAcβ1-2Manα1-3(**Neu5Acα2-6Galβ1-4GlcNAc**β1-2Manα1-6)Manβ1-4GlcNAcβ1-4GlcNAcβ-Sp12	8189
353	GlcNAcβ1-2Manα1-6(GlcNAcβ1-2Manα1-3)Manβ1-4GlcNAcβ1-4(Fucα1-6)GlcNAcβ-Sp22	7768
527	***Neu5Acα2-3Gal***β1-3GlcNAcβ1-2Manα-Sp0	6941
478	**Neu5Acα2-6Galβ1-4GlcNAc**β1-6(Galβ1-3GlcNAcβ1-3)Galβ1-4Glcb-Sp21	6901
315	**Neu5Acα2-6Galβ1-4GlcNAc**β1-2Manα1-3(GlcNAcβ1-2Manα1-6)Manβ1-4GlcNAcβ1-4GlcNAcβ-Sp12	6606
358	KDNa2-6Galβ1-4GlcNAc-Sp0	6532
333	**Neu5Acα2-6Galβ1-4GlcNAc**β1-3Galβ1-4GlcNAcβ1-3Galβ1-4GlcNAcβ-Sp0	6339
349	**Neu5Acα2-6Galβ1-4GlcNAc**β1-2Manα1-3Manβ1-4GlcNAcβ1-4GlcNAc-Sp12	6178
607	**Neu5Acα2-6Galβ1-4GlcNAc**β1-3Galβ1-4GlcNAcβ1-3Galβ1-4GlcNAcβ1-2Manα1-6(**Neu5Acα2-6Galβ1-4GlcNAc**β1-3Galβ1-4GlcNAcβ1-3Galβ1-4GlcNAcβ1-2Manα1-3)Manβ1-4GlcNAcβ1-4GlcNAcβ-Sp12	6161
479	***Neu5Aca2-3Gal***β1-4GlcNAcβ1-2Manα-Sp0	6154
51	**Neu5Acα2-6Galβ1-4GlcNAc**β1-2Manα1-3(**Neu5Acα2-6Galβ1-4GlcNAc**β1-2Manα1-6)Manβ1-4GlcNAcβ1-4GlcNAcβ-N(LT)AVL	5582
49	Neu5,9Ac2a2-6Galβ1-4GlcNAcβ-Sp8	5207
**B)**		
**Glycan No.**	**Glycan Name**	**Avg. RFU**
2	AGP-A (AGP ConA flowthrough)	52286.06
246	**Neu5Acα2-6Gal**β1-4GlcNAcβ–Sp8	49625.09
263	**Neu5Gcα2-6Gal**β1-4GlcNAcβ–Sp0	48932.24
250	**Neu5Acα2-6Gal**β1-4Glcβ–Sp8	48814.18
6	Transferrin	47533.26
248	**Neu5Acα2-6Galβ1-4GlcNAc**b1-3Galb1-4GlcNAcb-Sp0	47165.41
42	[6OSO3]Galβ1-4Glcβ–Sp0	34505.6
44	[6OSO3]Galβ1-4GlcNAcβ–Sp8	32444.86
247	**Neu5Acα2-6Galβ1-4GlcNAc**b1-3Galb1-4(Fuca1-3)GlcNAcb1-3Galb1-4(Fuca1-3)GlcNAcb-Sp0	30612.85
45	[6OSO3]Galb1-4[6OSO3]Glcb-Sp8	27055.41
245	**Neu5Acα2-6Galβ1-4GlcNAc**β–Sp0	26857.74
1	Alpha1-acid glycoprotein (AGP)	25869.33
43	[6OSO3]Galβ1-4Glcβ–Sp8	22740.63
86	GalNAcα1-3Galb–Sp8	21300.14
20	β-GalNAc–Sp8	20559.23
3	AGP-B (AGP ConA bound)	17780.79
72	Fucα1-2Galβ1-4GlcNAcβ–Sp8	15937.51
70	Fuca1-2Galb1-4GlcNAcb1-3Galb1-4GlcNAcb1-3Galb1-4GlcNAcb-Sp0	14916.26
69	Fucα1-2Galβ1-4GlcNAcβ1-3Galβ1-4GlcNAc–Sp0	13165.86
87	GalNAca1-4(Fuca1-2)Galb1-4GlcNAcb-Sp8	12866.52
242	**Neu5Acα2-6Gal**NAcα–Sp8	12071.82
90	GalNAcb1-3Gala1-4Galb1-4GlcNAcb-Sp0	11384.24
60	Fucα1-2Galβ1-3GalNAcβ1-4(***Neu5Acα2-3***)Galβ1-4Glcβ-Sp9	10906.21
120	Galβ1-3(Galβ1-4GlcNAcβ1-6)GalNAcα-Sp8	10546.55
150	Galβ1-4GlcNAcβ1-6(Galβ1-3)GalNAcα–Sp8	9937.06
251	**Neu5Acα2-6Gal**β–Sp8	9853.36
73	Fucα1-2Galβ1-4Glcβ–Sp0	9224.52
26	[3OSO3][6OSO3]Galb1-4[6OSO3]GlcNAcb-Sp0	9118.55
59	Fuca1-2Galb1-3GalNAcb1-4(***Neu5Aca2-3***)Galb1-4Glcb-Sp0	8069.79
74	Fucα1-2Galβ–Sp8	7769.91
122	Galb1-3(**Neu5Aca2-6**)GalNAca-Sp8	7693.01
10	α-GalNAc–Sp8	6840.72
40	[4OSO3]Galb1-4GlcNAcb-Sp8	6574.62
39	[4OSO3][6OSO3]Galb1-4GlcNAcb-Sp0	6514.83
241	**Neu5Acα2-6**(Galβ1-3)GalNAcα–Sp8	6184.61
87	GalNAca1-4(Fuca1-2)Galb1-4GlcNAcb-Sp8	5469.91
41	6-H2PO3Manα–Sp8	5447.01
249	**Neu5Acα2-6Gal**β1-4Glcβ–Sp0	5434.93
**C)**		
**Glycan No.**	**Glycan Name**	**Avg. RFU**
51	Manα1-6(Manα1-3)Manβ1-4GlcNAcβ1-4GlcNAcβ-Sp13	38866
352	Manα1-6(Galβ1-4GlcNAcβ1-2Manα1-3)Manβ1-4GlcNAcβ1-4GlcNAcβ-Sp12	37659
216	Manα1-6(Manα1-3)Manα1-6(Manα1-2Manα1-3)Manβ1-4GlcNAcβ1-4GlcNAcβ-Sp12	36933
347	Manα1-6(**Neu5Acα2-6Galβ1-4GlcNAc**β1-2Manα1-3)Manβ1-4GlcNAcβ1-4GlcNAc-Sp12	35539
212	Manα1-2Manα1-6(Manα1-3)Manα1-6(Manα1-2Manα1-2Manα1-3)Manβ1-4GlcNAcβ1-4GlcNAcβ-Sp12	35267
213	Manα1-2Manα1-6(Manα1-2Manα1-3)Manα1-6(Manα1-2Manα1-2Manα1-3)Manβ1-4GlcNAcβ1-4GlcNAcβ-Sp12	18208
217	Manα1-6(Manα1-3)Manα1-6(Manα1-3)Manβ1-4GlcNAcβ1-4GlcNAcβ-Sp12	15856
485	Manα1-6(Manα1-3)Manβ1-4GlcNAcβ1-4(Fuca1-6)GlcNAcβ-Sp19	12002
211	Manα1-6(Manα1-2Manα1-3)Manα1-6(Manα1-2Manα1-3)Manβ1-4GlcNAcβ1-4GlcNAcβ-Sp12	10800
417	Fuca1-2Galβ1-4(Fuca1-3)GlcNAcβ1-3GalNAca-Sp14	10154
477	Galβ1-3GlcNAcβ1-2Manα1-6(GlcNAcβ1-4)(Galβ1-3GlcNAcβ1-2Manα1-3)Manβ1-4GlcNAcβ1-4GlcNAcβ-Sp21	7265
50	Manα1-6(Manα1-3)Manβ1-4GlcNAcβ1-4GlcNAcβ-Sp12	6298
349	**Neu5Acα2-6Galβ1-4GlcNAc**β1-2Manα1-3Manβ1-4GlcNAcβ1-4GlcNAc-Sp12	6173
561	Gala1-3Galβ1-4GlcNAcβ1-2Manα1-6(Gala1-3Galβ1-4GlcNAcβ1-2Manα1-3)Manβ1-4GlcNAcβ1-4GlcNAc-Sp24	5565

A) Complex N-glycans for 10 SNA nodes (1002793, 1004421, 1004422, 1004780, 1004701, 1004702, 1004703, 1004704, 1004705 and 1004706) belonging to community 3 are listed. Majority of glycan nodes contain either show **Neu5Acα2-6Gal** or **Neu5Acα2-6Galβ1-4GlcNAc**, B) Four SNA (SNA-II) nodes (1004707, 1004708, 1004709 and 1004710) in the community 3 show preference for mainly mannose glycans. Only two glycans (glycan 347 and 349) possess the determinant **Neu5Acα2-6Galβ1-4GlcNAc,** C) less complex glycans for protein nodes 1000180, 1000181, 1000183, 1000184 and 1000725 in community 1. Few glycan show the determinant ***Neu5Acα2-3Gal*** (bold and italicized) which is known to inhibit glycan binding.

Compared to SNA-I nodes in community 3, five SNA-I nodes in community 1 (1000180, 1000181, 1000183, 1000184 and 1000725) interact with a smaller number of complex glycans (see **Table 2B**). Top 3 glycans possess either **Neu5Acα2-6Gal** or **Neu5Acα2-6Galβ1-4GlcNAc** and show RFU values greater than 40000. Two glycans from the second half of the table (glycans 60 and 59) show lower values of RFU because of the presence of the **Neu5Acα2-3Gal** sequence, which is known to decrease glycan binding. All these results are consistent with existing studies on the SNA specificity [27].

The 4 SNA-II nodes (1004707, 1004708, 1004709 and 1004710) in community 3 show preference for mainly mannose glycans or terminal **GlcNAcb1-4GlcNAcb**. Only two glycans (347 and 349) possess the determinant of **Neu5Acα2-6Galβ1-4GlcNAc** (**Table 2C**). In general, SNA-II is known to be Gal/GalNAc specific and is precipitated by glycoproteins, which consist of terminal GalNAc oligosaccharide chains [51]. Specifically, it shows higher affinity for D-GalNAc- and terminal N-acetyl-D-galactosaminyl disaccharides as compared to D-Gal. Conversely, the affinity exhibited by SNA-I for D-Gal and D-GalNAc- is identical [51]. However, SNA-I recognizes Neu5Acα2-6Gal [47] or Neu5Acα2-6Galβ1-4GlcNAc glycan sequence [27] with high specificity. Despite the differences in their glycan binding specificities, SNA-I and SNA-II share some similarities. For example, both lectins contain similar amino acid composition, while SNA-II contains more asparagine/aspartic acid, glycine and methionine residues [51]. Additionally, the carbohydrate-binding B-chains of both lectins show caspase-dependent apoptosis in different insect cell lines [52]. Considering their characteristic glycan binding specificities, SNA-I and SNA-II may play different functional roles in plants.

### Binding Specificities of ConA

Concanavalin A (ConA) binds to a variety of eukaryotic cells through specific interactions with saccharide-containing cellular receptors, and has been widely used as a molecular probe in studies of cell membrane dynamics and cell division [54]. ConA typically binds to glucosyl and mannosyl residues at the non-reducing termini of oligo- or polysaccharides [48], [49] and it can also bind to non-terminal mannosyl residues [50]. The current network contains sixteen nodes of ConA (1000158 and 1000165 in community 1; 1000356 and 1000699 in community 2; and 1004459, 1004460, 1004461, 1004462, 1004464, 1004465, 1004466, 1004467, 1004468, 1002791, 1004412 and 1004413 in community 3) which mainly interacts with mannose containing glycans.

All ConA interacting glycan nodes from community 1, 2 and 3 are shown in **Table 3A, 3B and 3C**, respectively. ConA interacting glycan nodes in community 1 are either mannose sugars or biantennary complex glycans such as transferrin and AGP-B. On the other hand, the ConA nodes in community 2 show preference for terminal glucose glycans.

**Table 3:** The table shows all types of glycans interacting with ConA protein nodes.

A)		
Glycan No.	Glycan Name	Avg. RFU
144	Manα1-2Manα1-6(Manα1-3)Manα1-6(Manα2Manα2Manα1-3)Manβ1-4GlcNAcβ1-4GlcNAcβ-N	31059
139	Manα1-3(Manα1-6)Manα–Sp3	23784
136	Mana1-2Mana1-3(Mana1-2Mana1-6)Mana-Sp9	23161
138	Mana1-3(Mana1-2Mana1-2Mana1-6)Mana-Sp9	17347
137	Mana1-2Mana1-3Mana-Sp9	14700
135	Mana1-2Mana1-2Mana1-3Mana-Sp9	14334
143	Mana1-6(Mana1-2Mana1-3)Mana1-6(Manα2Manα1-3)Manb1-4GlcNAcb1-4GlcNAcb-N	12786
145	Manα1-2Manα1-2Manα1-3(Manα1-2Manα1-3(Manα1-2Manα1-6)Manα1-6)Manβ1-4GlcNAcβ1-4GlcNAcβ-N	12581
112	α-D-Glc–Sp8	10407
75	Galβ1-4GlcNAcβ1-3Galβ1-4Glcβ–Sp8	8329
151	Neu5Gca2-3Galb1-4(Fuca1-3)GlcNAcb-Sp0	8141
59	Galβ1-3GalNAcβ1-4Galβ1-4Glcβ–Sp8	7380
113	mixed glycans: Man5-9-N–Sp1	6646
114	Manα1-6Manα1-3(Manα1-6Manα1-3)Manβ1-4GlcNAcβ1-4GlcNAcβ-N–Sp1	6600
146	Manα1-3(Manα1-6)Manβ1-4GlcNAcβ1-4GlcNAcβ–Sp2	6209
6	Transferrin	5981
130	Manα1-2Manα1-6(Manα1-3)Manα1-6(Manα2Manα1-3)Manβ1-4GlcNAcβ1-4GlcNAcβ-N–Sp1	5406
129	Manα1-6(Manα1-3)Manα1-6(Manα2Manα1-3)Manβ1-4GlcNAcβ1-4GlcNAcβ-N–Sp1	5259
3	AGP-B	5076
102	Fucα1-2Galβ1-4(Fucα1-3)GlcNAcβ–Sp8	5014
**B)**		
**Glycan No.**	**Glycan Name**	**Avg. RFU**
193	Manα1-2Manα1-6(Manα1-3)Manα1-6(Manα2Manα2Manα1-3)Manβ1-4GlcNAcβ1-4GlcNAcβ-N	52832
194	Manα1-2Manα1-2Manα1-3(Manα1-2Manα1-3(Manα1-2Manα1-6)Manα1-6)Manβ1-4GlcNAcβ1-4GlcNAcβ-Asn	52705
199	Man5-9mix-Asn	52238
198	Mana1-6(Mana1-3)Mana1-6(Mana1-3)Manb1-4GlcNAcb1-4 GlcNAcb-Asn	51705
196	Mana1-3(Mana1-2Mana1-2Mana1-6)Mana-Sp9	49576
192	Mana1-6(Mana1-2Mana1-3)Mana1-6(Manα2Manα1-3)Manb1-4GlcNAcb1-4GlcNAcb-Asn	48888
190	Mana1-2Mana1-3(Mana1-2Mana1-6)Mana-Sp9	44830
189	Mana1-2Mana1-2Mana1-3Mana-Sp9	43717
197	Manα1-6(Manα1-3)Manα1-6(Manα2Manα1-3)Manβ1-4GlcNAcβ1-4GlcNAcβ-N	40190
195	Manα1-3(Manα1-6)Manα–Sp9	38636
191	Mana1-2Mana1-3Mana-Sp9	35442
177	Glcα1-4Glcβ–Sp8	18139
179	Glcα1-6Glcα1-6Glcβ-Sp8	13465
178	Glcα1-4Glca–Sp8	12700
180	Glcb1-4Glcb-Sp8	6825
186	GlcAb1-6Galb-Sp8	6057
**C)**		
**Glycan No.**	**Glycan Name**	**Avg. RFU**
609	Neu5Aca2-6Galb1-4GlcNAcb1-3Galb1-4GlcNAcb1-2Mana1-6(Neu5Aca2-6Galb1-4GlcNAcb1-3Galb1-4GlcNAcb1-2Mana1-3)Manb1-4GlcNAcb1-4GlcNAcb-Sp12	11536
607	Neu5Aca2-6Galb1-4GlcNAcb1-3Galb1-4GlcNAcb1-3Galb1-4GlcNAcb1-2Mana1-6(Neu5Aca2-6Galb1-4GlcNAcb1-3Galb1-4GlcNAcb1-3Galb1-4GlcNAcb1-2Mana1-3)Manb1-4GlcNAcb1-4GlcNAcb-Sp12	26362
577	GlcNAcb1-3Galb1-4GlcNAcb1-3Galb1-4GlcNAcb1-2Mana1-6(GlcNAcb1-3Galb1-4GlcNAcb1-3Galb1-4GlcNAcb1-2Mana1-3)Manb1-4GlcNAcb1-4(Fuca1-6)GlcNAcb-Sp24	15181
576	Galb1-4GlcNAcb1-3Galb1-4GlcNAcb1-2Mana1-6(Galb1-4GlcNAcb1-3Galb1-4GlcNAcb1-2Mana1-3)Manb1-4GlcNAcb1-4(Fuca1-6)GlcNAcb-Sp24	37709
575	GlcNAcb1-3Galb1-4GlcNAcb1-2Mana1-6(GlcNAcb1-3Galb1-4GlcNAcb1-2Mana1-3)Manb1-4GlcNAcb1-4(Fuca1-6)GlcNAcb-Sp24	5650
561	Gala1-3Galb1-4GlcNAcb1-2Mana1-6(Gala1-3Galb1-4GlcNAcb1-2Mana1-3)Manb1-4GlcNAcb1-4GlcNAc-Sp24	7563
541	GlcNAcb1-3Galb1-4GlcNAcb1-2Mana1-6(GlcNAcb1-3Galb1-4GlcNAcb1-2Mana1-3)Manb1-4GlcNAcb1-4GlcNAcb-Sp25	40427
528	Gala1-3Galb1-3GlcNAcb1-2Mana-Sp0	5564
527	Neu5Aca2-3Galb1-3GlcNAcb1-2Mana-Sp0	19479
485	Mana1-6(Mana1-3)Manb1-4GlcNAcb1-4(Fuca1-6)GlcNAcb-Sp19	17039
484	Neu5Aca2-3Galb1-4GlcNAcb1-2Mana1-6(Neu5Aca2-3Galb1-4GlcNAcb1-2Mana1-3)Manb1-4GlcNAcb1-4(Fuca1-6)GlcNAcb-Sp24	34863
483	Neu5Aca2-6Galb1-4GlcNAcb1-2Mana1-6(Neu5Aca2-6Galb1-4GlcNAcb1-2Mana1-3)Manb1-4GlcNAcb1-4(Fuca1-6)GlcNAcb-Sp24	6282
477	Galb1-3GlcNAcb1-2Mana1-6(GlcNAcb1-4)(Galb1-3GlcNAcb1-2Mana1-3)Manb1-4GlcNAcb1-4GlcNAcb-Sp21	35401
476	GlcNAcb1-6(GlcNAcb1-2)Mana1-6(GlcNAcb1-2Mana1-3)Manb1-4GlcNAcb1-4(Fuca1-6)GlcNAcb-Sp24	10251
474	Fuca1-2Galb1-3(Fuca1-4)GlcNAcb1-2Mana1-6(Fuca1-2Galb1-3(Fuca1-4)GlcNAcb1-2Mana1-3)Manb1-4GlcNAcb1-4(Fuca1-6)GlcNAcb-Sp11-4(Fuca1-6)GlcNAcb-Sp19	8363
458	GalNAca1-3(Fuca1-2)Galb1-3GlcNAcb1-2Mana1-6(GalNAca1-3(Fuca1-2)Galb1-3GlcNAcb1-2Mana1-3)Manb1-4GlcNAcb1-4(Fuca1-6)GlcNAcb-Sp22	7281
456	Gala1-3(Fuca1-2)Galb1-3GlcNAcb1-2Mana1-6(Gala1-3(Fuca1-2)Galb1-3GlcNAcb1-2Mana1-3)Manb1-4GlcNAcb1-4(Fuca1-6)GlcNAcb-Sp22	31036
455	GalNAca1-3(Fuca1-2)Galb1-4GlcNAcb1-2Mana1-6(GalNAca1-3(Fuca1-2)Galb1-4GlcNAcb1-2Mana1-3)Manb1-4GlcNAcb1-4(Fuca1-6)GlcNAcb-Sp22	9449
428	Gala1-3(Fuca1-2)Galb1-4GlcNAcb1-2Mana1-6(Gala1-3(Fuca1-2)Galb1-4GlcNAcb1-2Mana1-3)Manb1-4GlcNAcb1-4(Fuca1-6)GlcNAcb-Sp22	19106
427	Fuca1-2Galb1-3GlcNAcb1-2Mana1-6(Fuca1-2Galb1-3GlcNAcb1-2Mana1-3)Manb1-4GlcNAcb1-4(Fuca1-6)GlcNAcb-Sp22	5130
425	Galb1-3GlcNAcb1-2Mana1-3(Galb1-3GlcNAcb1-2(Galb1-3GlcNAcb1-6)Mana1-6)Manb1-4GlcNAcb1-4GlcNAcb-Sp19	21266
424	Gala1-3(Fuca1-2)Galb1-4GlcNAcb1-2Mana1-3(Gala1-3(Fuca1-2)Galb1-4GlcNAcb1-2Mana1-6)Manb1-4GlcNAcb1-4(Fuca1-6)GlcNAcb-Sp22	32937
422	GlcNAcb1-2(GlcNAcb1-6)Mana1-6(GlcNAcb1-2Mana1-3)Manb1-4GlcNAcb1-4GlcNAcb-Sp19	10863
421	Fuca1-2Galb1-4GlcNAcb1-2Mana1-6(Fuca1-2Galb1-4GlcNAcb1-2Mana1-3)Manb1-4GlcNAcb1-4(Fuca1-6)GlcNAcb-Sp22	7369
418	GlcNAcb1-2Mana1-3(GlcNAcb1-2(GlcNAcb1-6)Mana1-6)Manb1-4GlcNAcb1-4GlcNAcb-Sp19	15686
405	Gala1-4Galb1-4GlcNacb1-2Mana1-6(Gala1-4Galb1-4GlcNacb1-2Mana1-3)Manb1-4GlcNacb1-4GlcNacb-Sp24	36858
404	Gala1-4Galb1-3GlcNacb1-2Mana1-6(Gala1-4Galb1-3GlcNacb1-2Mana1-3)Manb1-4GlcNacb1-4GlcNacb-Sp19	8992
399	Galb1-4GlcNAcb1-2Mana1-6(GlcNAcb1-2Mana1-3)Manb1-4GlcNAcb1-4GlcNAc-Sp12	37441
398	GlcNAcb1-2Mana1-6(Galb1-4GlcNAcb1-2Mana1-3)Manb1-4GlcNAcb1-4GlcNAc-Sp12	5577
396	Gala1-3Galb1-3(Fuca1-4)GlcNAcb1-2Mana1-6(Gala1-3Galb1-3(Fuca1-4)GlcNAcb1-2Mana1-3)Manb1-4GlcNAcb1-4GlcNAc-Sp19	36771
395	Gala1-3Galb1-3GlcNAcb1-2Mana1-6(Gala1-3Galb1-3GlcNAcb1-2Mana1-3)Manb1-4GlcNAcb1-4GlcNAc-Sp19	6293
394	GlcNAcb1-2Mana1-3(Galb1-4GlcNAcb1-2Mana1-6)Manb1-4GlcNAcb1-4GlcNAc-Sp12	28946
389	GlcNacb1-2Mana1-6(GlcNacb1-4(GlcNacb1-2)Mana1-3)Manb1-4GlcNacb1-4GlcNac-Sp21	6161
375	Gala1-3(Fuca1-2)Galb1-3GlcNAcb1-2Mana1-6(Gala1-3(Fuca1-2)Galb1-3GlcNAcb1-2Mana1-3)Manb1-4GlcNAcb1-4GlcNAcb-Sp20	15799
372	Gala1-3(Fuca1-2)Galb1-4GlcNAcb1-2Mana1-6(Gala1-3(Fuca1-2)Galb1-4GlcNAcb1-2Mana1-3)Manb1-4GlcNAcb1-4GlcNAcb-Sp20	8469
368	Gala1-3Galb1-4(Fuca1-3)GlcNAcb1-2Mana1-3(Gala1-3Galb1-4(Fuca1-3)GlcNAcb1-2Mana1-6)Manb1-4GlcNAcb1-4GlcNAcb-Sp20	21683
366	Fuca1-4(Galb1-3)GlcNAcb1-2Mana1-6(Fuca1-4(Galb1-3)GlcNAcb1-2Mana1-3)Manb1-4GlcNAcb1-4(Fuca1-6)GlcNAcb-Sp22	41588
365	Galb1-4GlcNAcb1-2(Galb1-4GlcNAcb1-4)Mana1-3(Galb1-4GlcNAcb1-2Mana1-6)Manb1-4GlcNAcb1-4GlcNAc-Sp21	12174
364	Gala1-3Galb1-4GlcNAcb1-2Mana1-6(Gala1-3Galb1-4GlcNAcb1-2Mana1-3)Manb1-4GlcNAcb1-4GlcNAcb-Sp20	42514
362	Fuca1-2Galb1-4GlcNAcb1-2Mana1-6(Fuca1-2Galb1-4GlcNAcb1-2Mana1-3)Manb1-4GlcNAcb1-4GlcNAcb-Sp20	5414
361	Fuca1-2Galb1-3GlcNAcb1-2Mana1-6(Fuca1-2Galb1-3GlcNAcb1-2Mana1-3)Manb1-4GlcNAcb1-4GlcNAcb-Sp20	21541
360	Mana1-3(Galb1-4GlcNAcb1-2Mana1-6)Manb1-4GlcNAcb1-4GlcNAcb-Sp12	33065
357	Fuca1-2Galb1-4GlcNAcb1-2Mana1-3(Fuca1-2Galb1-4GlcNAcb1-2Mana1-6)Manb1-4GlcNAcb1-4GlcNAcb-Sp20	27555
355	Galb1-3GlcNAcb1-2Mana1-6(Galb1-3GlcNAcb1-2Mana1-3)Manb1-4GlcNAcb1-4(Fuca1-6)GlcNAcb-Sp22	40353
354	Galb1-4GlcNAcb1-2Mana1-6(Galb1-4GlcNAcb1-2Mana1-3)Manb1-4GlcNAcb1-4(Fuca1-6)GlcNAcb-Sp22	10006
353	GlcNAcb1-2Mana1-6(GlcNAcb1-2Mana1-3)Manb1-4GlcNAcb1-4(Fuca1-6)GlcNAcb-Sp22	42532
352	Mana1-6(Galb1-4GlcNAcb1-2Mana1-3)Manb1-4GlcNAcb1-4GlcNAcb-Sp12	14973
349	Galb1-3GlcNAcb1-2Mana1-3(Galb1-3GlcNAcb1-2Mana1-6)Manb1-4GlcNAcb1-4(Fuca1-6)GlcNAcb-Sp22	38876
348	Galb1-4GlcNAcb1-2Mana1-3(Galb1-4GlcNAcb1-2Mana1-6)Manb1-4GlcNAcb1-4(Fuca1-6)GlcNAcb-Sp22	48582
347	GlcNAcb1-2Mana1-3(GlcNAcb1-2Mana1-6)Manb1-4GlcNAcb1-4(Fuca1-6)GlcNAcb-Sp22	41416
346	Neu5Aca2-6Galb1-4GlcNAcb1-2Mana1-6(Mana1-3)Manb1-4GlcNAcb1-4GlcNAc-Sp12	58408
328	Galb1-4(Fuca1-3)GlcNAcb1-2Mana1-6(Galb1-4(Fuca1-3)GlcNAcb1-2Mana1-3)Manb1-4GlcNAcb1-4GlcNAcb-Sp20	7604
327	Neu5Aca2-3Galb1-4GlcNAcb1-2Mana1-6(Neu5Aca2-6Galb1-4GlcNAcb1-2Mana1-3)Manb1-4GlcNAcb1-4GlcNAcb-Sp12	48590
322	Fuca1-3(Galb1-4)GlcNAcb1-2Mana1-3(Fuca1-3(Galb1-4)GlcNAcb1-2Mana1-6)Manb1-4GlcNAcb1-4GlcNAcb-Sp20	19979
321	GlcNAcb1-2Mana1-6(Neu5Aca2-6Galb1-4GlcNAcb1-2Mana1-3)Manb1-4GlcNAcb1-4GlcNAcb-Sp12	42652
320	Neu5Aca2-3Galb1-4GlcNAcb1-2Mana1-3(Neu5Aca2-3Galb1-4GlcNAcb1-2Mana1-6)Manb1-4GlcNAcb1-4GlcNAcb-Sp12	29652
319	Neu5Aca2-6Galb1-4GlcNAcb1-2Mana1-6(Neu5Aca2-3Galb1-4GlcNAcb1-2Mana1-3)Manb1-4GlcNAcb1-4GlcNAcb-Sp12	45597
317	Mana1-2Mana1-6(Mana1-2Mana1-3)Mana1-6(Mana1-2Mana1-2Mana1-3)Mana-Sp9	25444
316	Mana1-2Mana1-6(Mana1-3)Mana1-6(Mana1-2Mana1-2Mana1-3)Mana-Sp9	36802
315	Neu5Aca2-6Galb1-4GlcNAcb1-2Mana1-3(GlcNAcb1-2Mana1-6)Manb1-4GlcNAcb1-4GlcNAcb-Sp12	37868
314	Neu5Aca2-6Galb1-4GlcNAcb1-2Mana1-3(Galb1-4GlcNAcb1-2Mana1-6)Manb1-4GlcNAcb1-4GlcNAcb-Sp12	37828
313	Manα1-2Manα1-2Manα1-3(Manα1-2Manα1-6(Manα1-3)Manα1-6)Manα-Sp9	16574
312	Manα1-6(Manα1-3)Manα1-6(Manα1-3)Manβ-Sp10	6909
309	Neu5Aca2-6Galb1-4GlcNAcb1-2Mana1-6(GlcNAcb1-2Mana1-3)Manb1-4GlcNAcb1-4GlcNAcb-Sp12	41526
302	Neu5Aca2-6Galb1-4GlcNAcb1-2Mana1-6(Galb1-4GlcNAcb1-2Mana1-3)Manb1-4GlcNAcb1-4GlcNAcb-Sp12	6413
217	Mana1-6(Mana1-3)Mana1-6(Mana1-3)Manb1-4GlcNAcb1-4GlcNAcb-Sp12	38826
216	Mana1-6(Mana1-3)Mana1-6(Mana1-2Mana1-3)Manb1-4GlcNAcb1-4GlcNAcb-Sp12	28738
215	Mana1-2Mana1-2Mana1-6(Mana1-3)Mana-Sp9	34121
215	Mana1-2Mana1-2Mana1-6(Mana1-3)Mana-Sp9	34119
214	Mana1-6(Mana1-3)Mana-Sp9	39736
213	Manα1-6(Manα1-3)Manα1-6(Manα1-2Manα1-3)Manβ1-4GlcNAcβ1-4GlcNAcβ-Sp12	8657
212	Mana1-2Mana1-6(Mana1-3)Mana1-6(Mana1-2Mana1-2Mana1-3)Manb1-4GlcNAcb1-4GlcNAcb-Sp12	55882
211	Manα1-3(Manα1-6)Manα-Sp9	18045
210	Mana1-2Mana1-3Mana-Sp9	35979
209	Manα1-2Manα1-6(Manα1-3)Manα1-6(Manα1-2Manα1-2Manα1-3)Manβ1-4GlcNAcβ1-4GlcNAcβ-Sp12	7614
208	Mana1-2Mana1-2Mana1-3Mana-Sp9	37383
207	Manα1-2Manα1-3Manα-Sp9	6620
205	Mana1-3(Mana1-2Mana1-2Mana1-6)Mana-Sp9	24990
58	Neu5Aca2-6Galb1-4GlcNAcb1-2Mana1-6(Neu5Aca2-6Galb1-4GlcNAcb1-2Mana1-3)Manb1-4GlcNAcb1-4GlcNAcb-Sp24	8188
57	Neu5Aca2-6Galb1-4GlcNAcb1-2Mana1-6(Neu5Aca2-6Galb1-4GlcNAcb1-2Man-a1-3)Manb1-4GlcNAcb1-4GlcNAcb-Sp21	38425
56	Neu5Aca2-6Galb1-4GlcNAcb1-2Mana1-6(Neu5Aca2-6Galb1-4GlcNAcb1-2Mana1-3)Manb1-4GlcNAcb1-4GlcNAcb-Sp13	5338
55	Neu5Aca2-6Galb1-4GlcNAcb1-2Mana1-6(Neu5Aca2-6Galb1-4GlcNAcb1-2Mana1-3)Manb1-4GlcNAcb1-4GlcNAcb-Sp12	61880
54	Neu5Aca2-6Galb1-4GlcNAcb1-2Mana1-3(Neu5Aca2-6Galb1-4GlcNAcb1-2Mana1-6)Manb1-4GlcNAcb1-4GlcNAcb-Sp13	40848
53	GlcNAcb1-2Mana1-6(GlcNAcb1-2Mana1-3)Manb1-4GlcNAcb1-4GlcNAcb-Sp13	42923
52	Neu5Aca2-6Galb1-4GlcNAcb1-2Mana1-3(Neu5Aca2-6Galb1-4GlcNAcb1-2Mana1-6)Manb1-4GlcNAcb1-4GlcNAcb-Sp8	54802
51	Mana1-6(Mana1-3)Manb1-4GlcNAcb1-4GlcNAcb-Sp13	42505
50	Manα1-3(Manα1-6)Manβ1-4GlcNAcβ1-4GlcNAcβ-Sp13	8976
49	GlcNAcb1-2Mana1-3(GlcNAcb1-2Mana1-6)Manb1-4GlcNAcb1-4GlcNAcb-Sp13	5961
48	Mana1-3(Mana1-6)Manb1-4GlcNAcb1-4GlcNAcb-Sp13	37798

A) ConA interacting glycan nodes from community 1 are shown. These ConAs interact either with mannose nodes or biantennary complex glycans such as Transferrin and AGP-B, B) ConA interacting glycan nodes from community 2 are shown. They show preference for terminal glucose glycans, C) ConA nodes in community 3 show high preference for “N-glycan, high mannose” sugars.

In comparison to communities 1 and 2, the ConA nodes in community 3 show high preference for mannose containing sugars especially “N-glycan, high mannose” (**Table 3C**). These results agree with existing reports on ConA’s binding structure and specificity for mannose containing structures [55]-[57], in addition to the recognition of biantennary glycans, complex N-glycans [58] and terminal glucose [57].

Existing studies on SNA-I [47] and ConA [55]-[57] demonstrate the validity of the lectin-glycan interaction network and its detected community structure. Once a network is constructed, it is fairly easy to identify a lectin that explicitly binds to a certain glycan sequence by just selecting the lectin node of interest and its first neighbors in the network. The lectins in different communities show a dramatic difference in their glycan binding specificities. The current network-based approach should provide quick overall analysis and the use of glycan microarray data on the lectin-glycan interaction without time-consuming calculations.

### Community Detection of the Lectin-glycan Interaction

**Figure 4:**
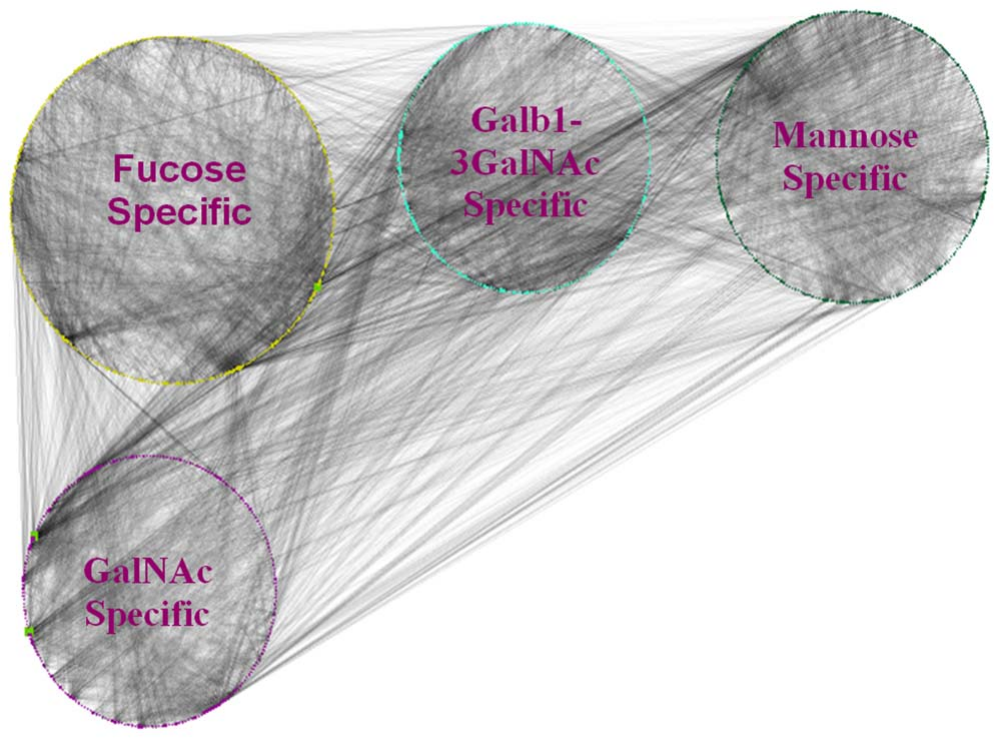
Graphical representation of four communities identified by Mod-CSA is shown. The figure provides an overall picture of the whole network with four main functional categories based on the p-value analysis.

**Figure 5:**
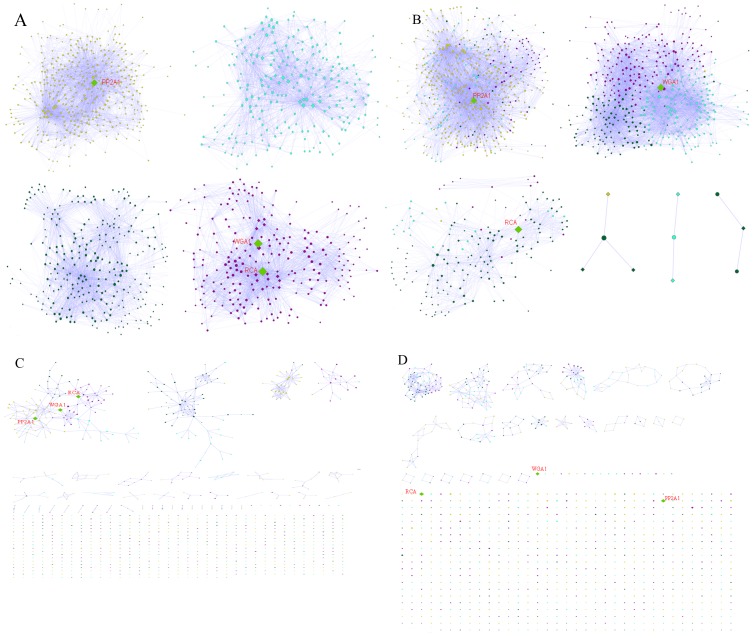
Communities generated by four methods are shown. (a) Mod-CSA generated communities are shown. In each community, glycans nodes are represented by circles whereas the protein nodes are shown as diamonds. From the figure it can be seen that all the nodes in a network have been assigned to a community. Community 1 has PP2A1 as hub node where as Community 4 has two hub nodes, WGA1 and RCA. (b) Greedy algorithm generated communities are shown. The nodes are color coded as per the Mod-CSA result. Each of the first three communities (community 1 to 3) contain a hub node where as communities 4-6 have only a few nodes.(c) MCODE generated communities are shown. Many nodes are not clustered, and the three hubs are grouped into one community. (d) MCL generated communities are shown many nodes are not clustered at all. Hub nodes are not clustered with any other nodes.

We performed community detection of the lectin-glycan interaction network by using Mod-CSA [28], and compared the results with existing methods such as MCL [34], [35], MCODE [36] and greedy algorithm [32], [38]. The number of identified communities and the modularity values obtained by various community detection algorithms are shown in **Table 4**, **Figure 4** and **Figure 5**.

**Table 4:** A summary of various clustering methods tested in this work.

Method	No. of Clusters	Modularity	Description
Mod-CSA	4	0.37	The conformational space annealing based modularityoptimization method.
Greedy	6	0.30	Fast greedy community detection algorithm.
MCODE	23	−0.04	Bader and Hogue algorithm for findingmodules in networks.
MCL	33	−0.81	Markov clustering algorithm from van Dongenthat uses random walks to simulate flow.

Mod-CSA outperforms the other popular clustering methods in terms of the modularity score.

From **Table** **4**, **Figure 4** & **Figure 5a–d** **,** it is clear that Mod-CSA [29] outperforms the other clustering methods in terms of the modularity score as well as the number of nodes left unclassified. The only method comparable to our modularity score of 0.37 obtained by Mod-CSA was the fast greedy algorithm [32], [38] with a modularity score of 0.30. The algorithm recognizes clusters by repetitively eliminating edges from the network and then checks again which nodes are still connected [59]. The method detected 6 communities with the largest community containing 223 protein nodes and 298 glycan nodes (community 1) whereas the three smallest communities consist of either 4 nodes (community 4) or 3 nodes (community 5 & 6) only (see **Figure 5b**).

To compare the biological significance of modules (communities) obtained by Mod-CSA and by the greedy algorithm, we calculated the numbers of statistically meaningful enriched clusters of lectins that bind to the same specific glycan. The glycan binding specificity of each protein node was identified either from the literature or from Uniprot database as described in the methods section, and the significance of each glycan specific clusters was assessed by calculating its p-value (p≤0.05). From **Table 5** **,** we observe that 44 statistically meaningful enriched clusters of lectins are identified with p-values ≤0.05. Whereas only 33 enriched clusters are identified by the greedy algorithm. This result suggests that many additional functionally related lectin clusters are identified by Mod-CSA, than detected by greedy algorithm.

**Table 5:** Lists of statistically meaningful enriched clusters (p≤0.05) of lectins binding to the identical glycan are shown.

Mod-CSA (Q = 0.366)				Community (Glay) (Q = 0.3)			
Cluster No.	No. of members	Reported Specificity	P-value	ClusterNo.	No. ofmembers	Reported Specificity	P-value
1	168	a-Linked terminal GalNAc	0.0055	1	223	a-Linked terminal GalNAc	0.0028
		Chitin oligomers, Sia	0.0109			Chitin oligomers, Sia	0.0006
		**Fuca1-2Galb1 -> or GalNAcb1 -> groups** **at their nonreducing terminals**	0.0347			Fuca1-2Galb1-3GalNAcb1-4(Neu5Aca2-3)Galb1-4Glcb OR Galb1-3GalNAcb1-4(Neu5Aca2-3)Galb1-4Glcb	0.0352
		Fuca1-2Galb1-3GalNAcb1-4(Neu5Aca2-3)Galb1-4Glcb OR Galb1-3GalNAcb1-4(Neu5Aca2-3)Galb1-4Glcb	0.0112			Fuca1-2Galb1-4GlcNAc	0.0001
		Fuca1-2Galb1-4GlcNAc	3.49E-06			Fuca1-6GlcNAc, Fuca1-3(Galb1-4)GlcNAc	0.0028
							
		**Fuca1-6GlcNAc (core fucose)**	0.0347			Fucose binding lectin	0.0028
		Fuca1-6GlcNAc, Fuca1-3(Galb1-4)GlcNAc	0.0004			***Galactose binding lectin***	0.0117
		Fucose binding lectin	0.0004			Galactose- and N-acetylgalactosamine-binding	0.0065
		Galactose- and N-acetylgalactosamine-binding	0.0147			Galb1-3GalNAc	0.0052
		Mannose binding lectin	0.0001			Galb1-3GalNAc, GalNAc	0.009
		**terminal N-acetylgalactosamine (GalNAc)**	0.0347			***Galα(1,3)Gal***	0.0352
2	98	Agalactosylated tri/tetra antennary glycans, GlcNAc	0.0011			High-mannose, Mana1-3Man	0.0403
		Chitin oligomers, Sia	5.27E-10			Mannose binding lectin	3.47E-06
							
		Galb1->3GalNAc-a-	4.40E-08			N,N'-diacetyllactosediamine(GalNAcβ1-4GlcNAc, LacdiNAc)	0.0369
							
		Galb1-3GalNAc	0.0007			***Siaa2-3Galb1-***	0.0151
		Galb1-3GalNAc, GalNAc	0.0021	2	190	(GlcNAcb1-4)n, Galb1-4GlcNAc	0.0257
		Mannose binding lectin	0.0207			Agalactosylated tri/tetra antennary glycans, GlcNAc	0.0257
		**N-acetylglucosamine and N-acetylneuraminic acid**	0.0068			Chitin oligomers, Sia	3.16E-07
							
3	147	Fuca1-6GlcNAc, a-D-Glc, a-D-Man	3.36E-05			Fuca1-2Galb1-4GlcNAc	0.0119
							
		Galb1-3GalNAc	0.0114			Fuca1-6GlcNAc, a-D-Glc, a-D-Man	0.0005
		High-mannose, Mana1-3Man	0.0223			Galactose binding lectin	0.0177
		High-mannose, Mana1-3Man, Mana1-6Man	0.0162			Galb1->3GalNAc-a-	4.17E-05
							
		High-mannose, Mana1-6(Mana1-3)Man	8.12E-07			High-mannose, Mana1-6(Mana1-3)Man	0.0485
							
		High-mannose, Mana1-6Man	0.0026			Mannose binding lectin	0.0196
		**Mana1-3(Mana1-6)Man, bi- and** **tri-antennary complex-type N-glycan, GalNAc**	0.0232			Siaa2-6Gal/GalNAc	0.0469
		**Manb Anywhere**	0.0232			Tri/tetra-antennary complex-type N-glycan	0.0184
		Mannose binding lectin	5.86E-06	3	93	High-mannose, Mana1-3Man	0.0092
							
		N-acetylglucosamine	0.0162			High-mannose, Mana1-3Man, Mana1-6Man	0.0105
		Siaa2-6Gal/GalNAc	0.0044			High-mannose, Mana1-6Man	5.32E-06
							
		Subterminal Mannose	0.0232			Mannose binding lectin	7.02E-14
4	100	(GlcNAcb1-4)n, Galb1-4GlcNAc	0.0013			N-acetylglucosamine	0.0105
		**a- or b-linked terminal GalNAc, GalNAca1-3Gal**	0.0003			Siaa2-6Gal/GalNAc	0.0049
		Agalactosylated tri/tetra antennary glycans, GlcNAc	0.0013			Subterminal Mannose	0.0058
		**Bi-antennary complex-type** **N-glycan with outer Gal and bisecting GlcNAc**	0.0056	4	4	NA	NA
		**Galb1-4GlcNAc**	0.0004	5	3	NA	NA
		**GalNAca1-3GalNAc, blood group A antigen**	0.0056	6	3	NA	NA
		**GalNAcb1-4GlcNAc, Galb1-3(-6)GalNAc**	0.0056				
		**GlcNAc oligomers, oligosaccharide** **containing GlcNAc and LacNAc**	0.0474				
		**GlcNAc trimers/tetramers**	0.0056				
		Mannose binding lectin	0.0003				
		N,N'-diacetyllactosediamine(GalNAcβ1-4GlcNAc, LacdiNAc)	0				
		**Siaa2-3Galb1-3(Siaa2-6)GalNAc**	0.0036				
		**Siaa2-3Galb1-4GlcNAc**	0.0377				
		Tri/tetra-antennary complex-type N-glycan	0.0234				

Communities generated by Mod-CSA and greedy algorithm are used. The statistical significance of each reported glycan binding lectin was calculated by hypergeometric distribution using p≤0.05. For each glycan listed in **Table S1**, interacting lectin nodes were identified to calculate the significance of the community structure determined in this study. The number of statistically significant glycan-specific groups according to Mod-CSA partitioning is 44 (p-value <0.05) while greedy algorithm provides only 33 groups. 15 glycan-specific groups generated by Mod-CSA but not by greedy algorithm are shown in bold, whereas 3 groups generated by greedy algorithm but not by Mod-CSA are shown in italic bold.

For example, the greedy algorithm failed to identify 15 glycan specific lectin clusters (shown in bold in **Table 5**) that were identified by Mod-CSA. On the contrary, 3 glycan specific clusters (shown in italic bold in **Table 5**) were not detected by Mod-CSA, which are found by the greedy algorithm result. Specifically, the greedy algorithm failed to identify all fucose specific lectins, while Mod-CSA [29] successfully detected almost all fucose specific lectins and grouped them in community 1. Similarly, the greedy algorithm identified only five mannose related specificities in community 3, which is the major mannose binding community detected by greedy algorithm. However, Mod-CSA recognized eight mannose related specificities in community 1.

We compared our method with other popular clustering algorithms such as MCODE [36] and MCL [34], [35]. MCODE method divided the network into a total of 23 clusters with the modularity score of −0.036. The largest cluster consists of 56 nodes whereas the smallest cluster contains only 4 nodes. However, only 3 clusters contain more than 10 protein nodes and they were further analyzed for enrichment of glycan specific lectin groups. The statistical analysis of these 3 clusters resulted in only 4 statistically meaningful lectin groups. From **Figure 5c**, we observe that a large number of single nodes (791) are not clustered into any groups. This is because MCODE identifies clusters of tightly connected nodes and does not intend to assign every node in the network to a cluster [59]. The main reason for this could be the fact that the MCODE algorithm is sensitive to noise in the network, particularly to false positive interactions [60]. Consequently, only a small number of strongly connected clusters are identified by MCODE and the rest of the nodes remain unclustered, which makes it hard to extract information from the network.

Among all four methods tested, the MCL algorithm performed worst in terms of its modularity value of −0.815. MCL detected 33 clusters with the largest cluster consisting of 340 nodes while the smallest cluster has 2 nodes (**Figure 5d**). Similar to MCODE, the MCL method detected only 3 clusters containing more than 10 protein nodes and many nodes (689) in the network were not assigned to any group, again making it difficult to interpret these unassigned nodes. Therefore, these unassigned nodes were left out for further analysis. The MCL method resulted in only 12 statistically significant glycan specific groups.

If the performances of MCL and MCODE are hindered by false positive interactions, MCL and MCODE may perform better with networks generated using only reliable data. To find out if the Mod-CSA method outperforms the other methods regardless of the amount of potentially false information, we performed the enriched cluster analysis on two additional networks generated using more stringent RFU criteria, RFU ≥10000 and RFU ≥20000 (see **Table S2**). The results remain same regardless of the RFU cutoff values used to generate the network. For example, the numbers of statistically significant glycan specific groups identified by Mod-CSA are 41 and 35 using RFU cutoff values of 10000 and 20000, respectively. However, the greedy algorithm provides 23 and 20 statistically significant glycan specific groups. Similarly, with the MCL method, 20 and 14 statistically significant glycan specific groups were identified (see **Table S3)**. Surprisingly, MCODE detected no statistically significant glycan specific lectin groups from more stringent networks.

**Figure 6:**
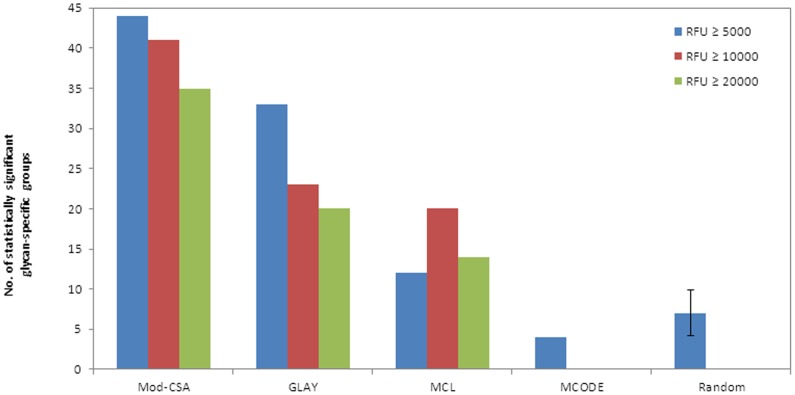
The number of statistically significant glycan-specific groups are shown for three networks generated with RFU cutoff values of 5000 (blue), 10000 (red), 20000 (green). The random clusterings are generated using the four community results of Mod-CSA, and the average and the standard deviation is calculated from 20 runs.

Finally? we compared the clusters obtained by Mod-CSA with random clusters. We divided the nodes into four random clusters, which have the same number of nodes with those detected by Mod-CSA. This process was iterated 20 times and the average number of statistically enriched glycan-specific groups detected by random clustering was compared with that by Mod-CSA. The maximum and minimum number of significantly enriched lectin groups was 11 and 1, respectively. On average, these 20 random permutations of clusters resulted in about 7 glycan-specific lectin groups having p-value ≤0.05 (see **Table S4**). A comparison of the number of significantly enriched lectin groups detected by the different clustering methods is shown in **Figure 6**. All these results demonstrate that Mod-CSA extracts more information than the other widely used clustering methods, and it can serve as a powerful tool for investigating the lectin-glycan interaction.

### The Optimal Community Structure of the Lectin-glycan Interaction Network

It has been shown that Mod-CSA can provide globally optimal modularity partitioning of a network containing up to 2000 nodes [31]. Since our lectin-glycan network has 1119 nodes, we believe that the Mod-CSA result corresponds to the optimal grouping of the network in terms of its modularity. The optimal modularity grouping of lectins and glycans results in 4 communities with the modularity score of 0.37. We attempted to explore the relationship between all nodes within the same community on the basis of structure and function of each lectin and the type of glycan binding specificity. Each lectin node was assigned with its known glycan binding specificity, and the statistical significance of their grouping was assessed by calculating its p-value (p≤0.05) (see **Table 5** and **Figure 4**). A brief description of each community is given below:

#### Community 1 (Fucose specific)

This is the largest community of the lectin-glycan network detected by Mod-CSA analysis and contains 168 protein nodes and 215 glycan nodes, respectively. This community is dominated by protein nodes with fucose specific lectins, such as ulex europaeus agglutinin I (UEA-I), aleuria aurantia lectin (AAL), ralstonia solanacearum lectin (RSL), etc. The fucose binding sites of RSL are very similar to those of previously reported five fucose-binding sites of AAL [61]. Fucose-containing xyloglucans are known to promote signaling consequences on plant tissues [62]. The other types of overrepresented lectins in this community have specificity for Galactose- and N-acetylgalactosamine binding with cell adhesion as their main function. The most common protein domains correspond to these galactose specific lectins are H_lectin (PFAM ID: PF09458) domain, which is involved in self/non-self recognition of cells through binding with carbohydrates [63], and Galactose-binding domain-like domain known as Discoidin domain (PFAM ID: PF00754), which is found in many blood coagulation factors. The galactose specific lectins in this community include agglutinin from Helix pomatia, Discoidin I and Discoidin II from Dictyostelium discoideum (Slime mold). Additionally, the unannotated lectins in this cluster such as 6RG, Tap1, Mubin1 show specificity for galactose or fucose sugars (see Table S5), which strongly indicates that these proteins are related to cell adhesion.

This community contains the top hub PP2A1 (1001943) with the largest node degree of 257. The other three PP2A1 nodes (1002090, 1002091 and 1002092) belong to community 2. The list of unique glycans that interact with these PP2A1 nodes are summarized in Table S6. From this table it can been seen that PP2A1 nodes show specificity for a diverse range of glycans such as GlcNAc, high-mannose *N*-glycans and sialic acid containing glycans. Recently, Beneteau et al., (2010) [64] in their glycan array experiments have shown that PP2A1 binds to different types of carbohydrates. This indicates the possibility that the phloem PP2 lectin plays roles in numerous functions, recognizing either endogenous glycoproteins or glycosylated receptors of pathogens. This diversity in glycan binding by PP2A1 could be attributed to the presence of several carbohydrate-binding sites in PP2A1 [64].

#### Community 2 (Galb1-3GalNAc specific)

This is the smallest community with 98 protein nodes and 133 glycan nodes. Community 2 is rich in N-acetylglucosamine and N-acetylgalactosamine binding lectins such as Wheat Germ Agglutinin (WGA), Griffonia simplicifolia II (GS-II), and Sclerotium rolfsii lectin (SRL). WGA belongs to a highly conserved family of chitin-binding lectins from cereals (*Gramineae*), such as rye, barley, rice and wheat [65]. Chitin, a polymer of β-1,4-N-acetylglucosamine is present in the cell wall of many fungi, in the exoskeleton and digestive tract of some insects, and in some nematodes [66]. Similarly, GS-II, also an N-acetylglucosamine-specific legume lectin, has insecticidal activity against cowpea weevil [67]. In contrast to WGA and GS-II, SRL displays strong binding to O-linked galactose-beta-1,3-N-acetylgalactosamine, disaccharide (Thomsen Friedenreich antigen) similar to Agaricus bisporus lectin [68]. Similarly, the other N-acetylgalactosamine specific lectins in this group are involved in the binding of T-antigen structure Gal-beta1,3-GalNAc e.g. Agglutinin alpha chain (Jacalin alpha chain) from Artocarpus integer (Jack fruit) and Agglutinin alpha chain (MPA) from Maclura pomifera (Osage orange). Unannotated protein nodes are represented by lectins such as Protein PHLOEM PROTEIN 2-LIKE A1 (PP2A1) from Arabidopsis thaliana and Codium fragile lectin (CFT) from Codium fragile **[**(Dead man's fingers) (Green alga)]**.** PP2A1 is known to interact with diverse types of carbohydrates and may be involved in numerous recognition functions [64]. On the other hand, CFT shows preference for the a-anomer of GalNAc and recognizes GalNAca1 sequences as well as high affinity for the Forssman pentasaccharide and for Galb1->3GalNAc-a- [69], which is one of the overrepresented (p-value <0.05) glycan specific group in this community. Lists of unique glycans for PP2A1 and CFT nodes are summarized in **Table S7**.

#### Community 3 (Mannose specific)

Protein nodes in this group are dominantly mannose binding lectins and nine out of twelve statistically significant glycan groups are mannose specific. Many members of these mannose specific lectins have B_lectin (PFAM ID: PF01453) structural domain. The members of this family are mannose specific and belong to Bulb lectin super-family (Amaryllidaceae, Orchidaceae and Aliaceae).For example, Galanthus nivalis agglutinin (GNA), a mannose-specific lectin from snowdrop bulbs, is a tetrameric member of the family of Amaryllidaceae lectins that exhibit antiviral activity towards HIV [70]. Other mannose binding lectins in this group have Lectin_legB (PFAM ID: PF00139) structural domain and require metal ions like Ca and Mn ions for carbohydrate binding and cell-agglutinating activities. Examples include ConA and Garden pea lectin. The group also includes various high mannose binding lectins such as Hippeastrum hybrid lectin (HHL), Narcissus psuedo-narcissus agglutinin (NPA), Salt stress-induced protein, Allium sativum agglutinin (ASA), etc. Another mannose binding lectin in this group which has an antiviral activity is Cyanovirin-N (CV-N). The antiviral activity of CV-N is mediated through specific interactions with the viral surface envelope glycoproteins gp120 and gp41, as well as to high-mannose oligosaccharides found on the HIV envelope [71].

Other lectins that were grouped in this community for which we could not find the reported glycan specificity include Arum maculatun agglutinin (AMA), Caragana arborescens agglutinin (CAA), Colchicum autumnale lectin (CA), and Arisaema helleborifolium schott lectin (AHL). All these lectins also show high specificity for mannose sugars (**Table S8**). Overall the community consists of 147 protein nodes and 124 glycan nodes.

#### Community 4 (GalNAc specific)

From **Table 5** it can be observed that this community is enriched in GalNAc specific lectins such as Datura stramonium agglutinin (DSA), Soybean agglutinin (SBA), Vicia villosa agglutinin (VVA), Bauhinia purpurea lectin (BPL), etc. These galactose specific lectins may play a significant role in cell-agglutinating activities e.g. VVA (Lectin B4) from Vicia villosa (Hairy vetch). Another galactose-specific lectin in this group is a legume lectin known as Erythrina cristagalli lectin (ECL) [72]. Although its function in the legume is unknown, it has been shown that ECL possesses hemagglutinating activity and it is believed to be mitogenic for human T lymphocytes [73]. A large number of plant and fungal proteins (e.g. solanaceous lectins of tomato and potato, plant endochitinases, the wound-induced proteins: hevein, win1 and win2, and the Kluyveromyces lactis killer toxin alpha subunit) that bind N-acetylglucosamine contain chitin-binding domain (PFAM ID: PF00187). These proteins might function as a defence against chitin containing pathogens, e.g. Chitin-binding lectin 1 of Solanum tuberosum (Potato). This community also includes lectins such as Macrolepiota procera agglutinin (MPA) and Laccaria bicolor lectin both of which show high specificity for complex GalNAc glycans (**Table S9**). This community consists of 100 protein and 134 glycan nodes.

Additionally, this community includes 2 out of three hub nodes identified in the lectin-glycan array network. One of the hubs represent protein node (1004763) for wheat germ agglutinin (WGA) from Triticum vulgaris (wheat), whereas the second node (1004668) represents Ricinus communis agglutinin (RCA) from Ricinus communis (castor bean). WGA is a stable homodimer protein and exhibits specificity for N-acetylneuraminic acid and N-acetylglucosamine (GlcNAc) sugars. The glycans for WGA hub node are summarized in **Table S10** and it can be observed that almost all these glycans have GlcNAc group, while few others contain N-acetylneuraminic acid. Each monomeric unit of WGA consists of four domains (A–D) which can be further classified into “primary” (B and C domains) and “secondary” (A and D domains) binding sites showing dissimilar affinities for GlcNAc containing moieties [74]. These structural characteristics and the closeness of binding sites make WGA a worthy candidate to explore multivalent protein-carbohydrate interactions and to assess the impact of structural modifications of glycoclusters [75]. These multivalent interactions are favorable as compared to monomeric ones and are frequently employed by nature to control an array of diverse biological processes [76].

RCA as well as ECL recognize carbohydrate chains with non-reducing terminal β-d-galactose (Galβ) and show preference to Galβ1-4GlcNAc instead of Galβ1-3GlcNAc sequence [77], [78]. The diverse types of glycans including Galβ1-4GlcNAc that interact with RCA hub node are listed in **Table S11**. The table also shows many Neu5Aca2-6Galb1 sugars having large RFU values.?RCA is a glycoprotein from seeds of castor plants and one of the most important applied lectins that have been widely used as a tool to study cell surfaces and to purify glycans [79]. RCA promotes binding and agglutination of polysaccharides and glycoproteins in addition to liposomes and micelles containing glycolipids with galactosyl residues [80], [81]. Furthermore, the specificities of interactions of RCA with neutral and sialylated oligosaccharides have been well established and is consistent with our results as summarized in **Table S11** [82].

The current community-based network study of the lectin-glycan microarray data provides not only a quick and systematic analysis of lectin specificities, but also global organization and grouping of biologically related lectins along with their binding partners (glycans). Such information will be vital to identify lectins that bind to particular glycan structures or to catalogue lectins according to the similarity in specificities. Another important significance of the community-based network analysis is the identification of a novel lectin and the initial guess about its specificity. For this, a sequence database should be constructed for each community identified and a target lectin under investigation should be fed into the databases to get an idea about the structural/functional role of the query lectin and the type of glycans it might bind to. This approach will be more practical when the communities have a large number of different lectins and might help in determining the glycan binding nature of a given lectin. There are many network-based protein function prediction methods along with approaches utilizing structural or sequence information of proteins. Recently, when dealing with a protein-protein-interaction network, it has been shown that more accurate protein function prediction results were obtained by modularity based community detection of the network. The current study provides the first attempt to study lectin-carbohydrate interactions via community detection of a network.

## Conclusion

We have constructed a bipartite lectin-glycan interaction network from the collection of glycan microarray data. The network itself provides a quick and global view of the lectin-glycan interaction from which hub proteins are identified. We find that the hub proteins match well with the characteristics of known biological relevance. Using Mod-CSA, a recently developed efficient community detection method, 4 modules are identified. The clustering results are shown to be biologically more meaningful than those obtained by other widely used methods. Most significantly, 44 statistically significant glycan specific groups are identified including fucose and mannose binding ones, some of which could not be detected by alternative methods. Even with more strict RFU cut-offs, clusters generated by Mod-CSA provide consistently better results as compared to other methods. We provide overall analysis of 4 communities identified in the lectin-glycan microarray network. We also show how multiple lectins from the same plant, such as *Sambugus nigra* (SNA-I and SNA-II) are grouped into different communities based on their glycan binding specificities. The network study provides a framework to get a broad picture of data containing many interacting components. These capabilities of a community-based network analysis allow researchers to explore, analyze and compare a variety of proteins and glycans within the context of modules/communities identified in the network. We expect that this will trigger interest in the prediction of protein-carbohydrate interactions using biological networks and will have wider applications as additional glycan binding proteins are identified. The method can also be applied to study other types of lectins as well as other interaction networks.

## Supporting Information

Table S1 **List of all protein nodes, their clusters and reported specificity in the lectin-glycan network.**
(XLS)

Table S2 **The list of meaningful glycan-specific groups and their P-values detected by Mod-CSA and greedy algorithm (GLAY) at RFU ≥10000 and RFU ≥20000.**
(XLS)

Table S3 **The list of meaningful glycan-specific groups and their P-values detected by MCL and MCODE at RFU ≥5000, RFU ≥10000 and RFU ≥20000.**
(XLS)

Table S4 **List of randomly identified statistically significant glycan-specific groups.**
(DOCX)

Table S5 **List of unique galactose and fucose sugars that interact with unannotated 6RG, Tap1, and Mubin at RFU ≥5000 in the lectin-glycan network.**
(XLS)

Table S6 **List of diverse glycans that interact with the hub PP2A1 at RFU ≥5000 in the lectin-glycan network.**
(XLS)

Table S7 **Lists of unique glycans for PP2A1 and CFT at RFU ≥5000 in the lectin-glycan network.**
(XLS)

Table S8 **List of unique glycans for unannotated lectins Arum maculatun agglutinin (AMA), Caragana arborescens agglutinin (CAA), Colchicum autumnale lectin (CA), and Arisaema helleborifolium schott lectin (AHL) that show high specificity for mannose sugars at RFU ≥5000 in the lectin-glycan network.**
(XLS)

Table S9 **List of complex glycans that show high specificity for lectins such as Macrolepiota procera agglutinin (MPA) and Laccaria bicolor lectin.**
(XLS)

Table S10 **List of diverse glycans that interact with the hub WGA at RFU ≥5000 in the lectin-glycan network.**
(XLS)

Table S11 **List of diverse glycans that interact with the hub RCA at RFU ≥5000 in the lectin-glycan network.**
(XLS)
