# Supplementary material for: Community-Based Network Study of Protein-Carbohydrate Interactions in Plant Lectins Using Glycan Array Data
Source: PLoS One. 2014 Apr 22;9(4):e95480. doi: 10.1371/journal.pone.0095480 (PMC3995809; doi:10.1371/journal.pone.0095480)
Supplement: Table S4 — List of randomly identified statistically significant glycan-specific groups. (DOCX) [file pone.0095480.s004.docx]

**Supplementary Table 4**

|  | **Randomly Identified Statistically Significant Glycan Specific groups** | | | | |  |  |
| --- | --- | --- | --- | --- | --- | --- | --- |
| **Iteration No.** | **Comm1** | **Comm2** | **Comm3** | **Comm4** | **Total** | **Mean** | **Std** |
| **R1** | 3 | 2 | 2 | 0 | 7 | 1.75 | 1.26 |
| **R2** | 1 | 1 | 2 | 1 | 5 | 1.25 | 0.50 |
| **R3** | 0 | 2 | 2 | 1 | 5 | 1.25 | 0.96 |
| **R4** | 0 | 4 | 3 | 2 | 9 | 2.25 | 1.71 |
| **R5** | 1 | 0 | 1 | 2 | 4 | 1 | 0.82 |
| **R6** | 3 | 1 | 3 | 1 | 8 | 2 | 1.15 |
| **R7** | 1 | 1 | 2 | 2 | 6 | 1.5 | 0.58 |
| **R8** | 4 | 2 | 1 | 1 | 8 | 2 | 1.41 |
| **R9** | 3 | 1 | 2 | 2 | 8 | 2 | 0.82 |
| **R10** | 3 | 0 | 3 | 0 | 6 | 1.5 | 1.73 |
| **R11** | 4 | 3 | 3 | 0 | 10 | 2.5 | 1.73 |
| **R12** | 1 | 0 | 0 | 0 | 1 | 0.25 | 0.50 |
| **R13** | 2 | 6 | 1 | 2 | 11 | 2.75 | 2.22 |
| **R14** | 3 | 5 | 2 | 1 | 11 | 2.75 | 1.71 |
| **R15** | 2 | 3 | 1 | 4 | 10 | 2.5 | 1.29 |
| **R16** | 2 | 2 | 2 | 5 | 11 | 2.75 | 1.50 |
| **R17** | 1 | 3 | 0 | 0 | 4 | 1 | 1.41 |
| **R18** | 0 | 0 | 0 | 3 | 3 | 0.75 | 1.50 |
| **R19** | 1 | 1 | 1 | 4 | 7 | 1.75 | 1.50 |
| **R20** | 2 | 2 | 1 | 1 | 6 | 1.5 | 0.58 |
| **Grand Total** | **37** | **39** | **32** | **32** | **140** |  |  |
| **Mean** | **1.85** | **1.95** | **1.6** | **1.6** | **7** |  |  |
| **Std** | **1.27** | **1.67** | **0.99** | **1.47** | **2.85** |  |  |
